# Transcriptomic analysis revealed the protective role of genistein against oxidized fish oil-induced oxidative stress and inflammatory responses in turbot (*Scophthalmus maximus*)

**DOI:** 10.3389/fphys.2026.1900021

**Published:** 2026-08-04

**Authors:** Lin Yang, Xiaoqian Zhou, Junfeng Hu, Ziyu Jia, Shuyun Jiang, Dan Xu, Zhijie Dan

**Affiliations:** 1 Jiangsu Key Laboratory of Marine Bioresources and Environment, Jiangsu Ocean University, Lianyungang, China; 2 School of Ocean, Yantai University, Yantai, China; 3 Jiangsu Institute of Marine Resources Development, Lianyungang, China

**Keywords:** hepatic oxidative stress, hspd1, inflammatory response, msrb2, transcriptomics, WGCNA

## Abstract

**Introduction:**

Extensive investigations have been conducted across mammalian species to explore the redox-modulating and inflammation-resolving properties of genistein and the mechanisms involved; however, research on the functions of this compound in aquatic species stay insufficient. Therefore, the present research was designed to determine whether oxidative liver injury and inflammatory immune reactions in turbot (*Scophthalmus maximus*) fed an oxidized fish oil diet could be ameliorated by genistein supplementation.

**Methods:**

In this study, three dietary treatments were established: a fresh fish oil group (FFO), an oxidized fish oil group (OFO) in which fresh oil was completely replaced by oxidized oil, and a GEN40 group, where 40 mg/kg genistein was supplemented to the OFO basal diet. Juvenile turbot with an initial mean body mass of 10.00 ± 0.03 g were used in this study, and the feeding trial lasted 12 weeks.

**Results:**

As revealed by the results, hepatic mitochondrial impairment was induced in turbot by oxidized fish oil ingestion, as reflected by reduced cristae number, cristae disorganization, and concentric cristae formation, while a significant increase in hepatic superoxide anion abundance was also noted (*P* < 0.05). These mitochondrial alterations were partially alleviated by genistein supplementation, with which a marked decrease in superoxide anion levels was concurrently observed (*P* < 0.05). Moreover, the hepatic mRNA levels linked to inflammation, oxidative stress, and apoptosis were markedly upregulated in the OFO group (*P* < 0.05), yet declined in the GEN40 group (*P* < 0.05). Transcriptomic analysis revealed that after genistein supplementation in oxidized fish oil-based diets, the DEGs were predominantly enriched in Kyoto Encyclopedia of Genes and Genomes (KEGG) pathways such as the Peroxisome Proliferator-Activated Receptor (PPAR), calcium signaling, and adrenergic signaling pathways. It was further revealed by Gene Ontology (GO) enrichment analysis that the OFO group chiefly suppressed lipid catabolism and signal transduction, while activating the immune response. Compared with the FFO group, the genistein-treated group downregulated lipid metabolism and upregulated substance transport processes. Relative to the OFO group, it downregulated signal transduction and upregulated RNA-related catalytic activities. Moreover, methionine-S-sulfhydryl reductase B2 (*msrb2*) and heat shock protein 60 (*hspd1*) were identified as hub genes through Weighted Gene Co-expression Network Analysis (WGCNA) and were associated with the genistein-mediated alleviation of hepatic oxidative and inflammatory stress that was triggered by oxidized fish oil in turbot.

**Discussion:**

The above results confirmed that adding 40 mg/kg of genistein to an oxidized fish oil-based diet significantly alleviated hepatic oxidative stress and inflammatory injury mediated by the oxidized fish oil; besides *msrb2* and *hspd1* possibly served a critical function in this process.

## 1 Introduction

Fish oil was provided as the essential source of lipids in aquacultural feeds and markedly strengthens the growth, health, and reproductive performance of aquatic species (Fatima et al., 2019) but because of its highly unsaturated nature, fish oil exhibits a high propensity for oxidation during processing and preservation, producing primary lipid oxidation products along with secondary oxidative compounds, aldehydes, alcohols, and other volatiles (Laguerre et al., 2007). Furthermore, the oxidation and deterioration of fish oil not only reduce the nutritional value of the feed, but also the harmful metabolites produced can also trigger severe oxidative damage and inflammation when ingested by aquatic animal species, thereby causing serious harm to their health (Wang et al., 2025). Myriad investigations have established that oxidized oil can attenuate growth (Huang et al., 2006; Gao et al., 2012; Sutton et al., 2006), disrupted lipid metabolism (Hasanpour et al., 2017; Dong et al., 2014; Liu et al., 2019; Zhang et al., 2022; Rahimnejad et al., 2021), impair oxidative defense capacity (Liang et al., 2019; Chen et al., 2019), and cause intestinal injury in fish (Zhuo et al., 2018). Although artificial antioxidants including butylated hydroxytoluene (BHT) and ethoxyquin (EQ) can markedly inhibit lipid oxidation, nevertheless, their possible cellular toxicity may cause harm to fish and result in environmental contamination (Euler and Pietsch, 2018). Accordingly, there is a pressing requirement for toxicity-free, effective, and pleiotropic natural antioxidants to alleviate the detrimental effects resulting from oxidized fish oil in aquafeeds.

Soy isoflavones (ISF), belonging to the phytoestrogen family, are secondary metabolites that accumulate naturally as leguminous plants develop (Lee et al., 2006); under natural conditions, these compounds typically exist in glycoside-bound forms. Their glycoside forms possess a variety of biological functions (Luo et al., 2023). Genistein, as one of its most biologically active aglycones (Mai et al., 2012), plays a positive role in anti-inflammatory, antibacterial, anticancer, antioxidant, immunomodulatory, and cell cycle regulatory activities (Nazari-Khanamiri and Ghasemnejad-Berenji, 2021). Researchers have found that genistein possesses antioxidant properties; in mammals, genistein can inhibit the initiation of oxygen free radicals or ROS, significantly reducing the levels of malondialdehyde (MDA) in tissues that have risen due to oxidative stress (Tuli et al., 2019); other studies had demonstrated that genistein exerts a protective role against neurotoxicity, oxidative stress, and chronic alcoholic liver injury, while its anti-inflammatory action was mediated through the inhibition of nuclear factor kappa-B (NF-κB), along with the suppression of pro-inflammatory mediators such as prostaglandins (PGs) and the enzyme Inducible nitric oxide synthase (iNOS) (Yu et al., 2021); Additionally, the literature had documented that a diet designed to reduce lipid oxidative spoilage can lower MDA levels in the livers of common carp (*Cyprinus carpio*) originating from the Yellow River, and reduce hepatic lipidosis and inflammation (Witayavanitkul et al., 2020). Earlier investigations revealed that adding 500 mg/kg of genistein to high-sugar or high-fat diets promoted fat metabolism in carp, enhanced the antioxidant enzyme activities, such as glutathione peroxidase (*gpx*) and superoxide dismutase (*sod*); reduced MDA contents, also improves the body’s antioxidant capacity (Yang et al., 2023); However, adding high doses of genistein to turbot feed could reduce digestive enzyme activity and cause some damage to the intestinal tissue structure (Guo et al., 2014). In summary, genistein serves a key function in alleviating inflammatory and oxidative burden responses in animals. However, the molecular mechanisms by which genistein mitigates inflammatory responses and oxidative damage in fish livers remain unclear.

The turbot (*Scophthalmus maximus*) is a typical piscivorous fish widely cultivated in China and Europe (Dou et al., 2024). This species requires a dietary lipid level of approximately 10% and crude protein content above 50% (Luo et al., 2022; Liu et al., 2015). Because of its high protein requirement, turbot has emerged as an ideal model organism to explore alternative protein resources for marine aquaculture (Burel et al., 2000; Liu et al., 2014). Meanwhile, oxidized fish oil exerts detrimental effects on turbot, including the promotion of oxidative stress and the perturbation of intestinal microbiota homeostasis (Wang et al., 2025). In a previous study by our group, genistein was demonstrated to alleviate lipopolysaccharide (LPS)-induced hepatic oxidative damage and inflammation in juvenile turbot (Zhou et al., 2024). Moreover, genistein supplementation at 40 mg/kg was proven to be particularly effective in attenuating oxidized fish oil-induced oxidative injury and inflammation, while also ameliorating hepatic lipid metabolism, ameliorating intestinal barrier impairment, and optimizing gut microbiota in juvenile turbot (Yu et al., 2020). Therefore, this research explored the alleviative effects of genistein supplementation in OFO diets on hepatic oxidative burden and inflammatory responses in turbot caused by dietary oxidized lipids, and preliminarily clarified the potential regulatory mechanisms and key functional genes based on transcriptome sequencing. The study aimed to clarify the molecular mechanism by which genistein protected against oxidized fish oil-induced hepatic damage in turbot, provide a theoretical reference for applying flavonoids as plant-derived bioactive substances to improve oxidative injury and hepatic inflammatory responses in aquatic organisms, and further promote healthy fish culture.

## 2 Methods

### 2.1 Preparation of test diets

Oxidized fish oil with a peroxide value of 231 mmol/kg was prepared according to Yin et al. (2018). Three dietary treatments were established: a fresh fish oil group (FFO), an oxidized fish oil group (OFO) in which fresh oil was completely replaced by oxidized oil, and a GEN40 group, where 40 mg/kg genistein was supplemented to the OFO basal diet. The supplemental dosage of genistein was set based on Yu et al. (2020). Dietary formulations are presented in the **Supplementary Materials**.

### 2.2 Rearing experiment

After a two-week acclimatization period and a 24-hour fasting period, the turbot were individually weighed, followed by a 12-week feeding and growth experiment. A total of 180 juvenile fish were randomly distributed among 9 plastic rearing tanks (160 liters per tank). The experimental design was as follows: during the cultivation period, the culture water consisted of self-prepared seawater in a recirculating system; the rearing temperature was kept at 21°C, the water Salinity was regulated to stay within 28-30; the level of oxygen concentration was maintained at 7 mg/L or higher; and ammonia-nitrogen concentrations were remained under 0.20 mg/L. Daily feeding was conducted twice, at 08:00 and 18:00, until they stopped feeding.

### 2.3 Tissue sampling

Following the 12-week feeding experiment, a 24 h period of feed deprivation was imposed prior to sampling. Before tissue collection, eugenol was used to anesthetize the turbot. For blood collection, the syringe was inserted at a 45° angle slightly below the lateral line near the anal fin. The needle was gently advanced until it contacted the vertebra, then angled slightly to enter the blood vessel, and the blood was withdrawn. Blood samples were subjected to centrifugation (3000 rpm, 10 min, 4°C). Then, the supernatant (serum) was carefully separated to avoid perturbing the underlying red blood cell pellet. For each dietary treatment, several fish were randomly selected from the tanks. Six of them were used for quantitative analysis and enzyme activity assays, while another five were assigned to transcriptomic sequencing, Transmission Electron Microscope (TEM) and Reactive Oxygen Species (ROS) detection. For the latter, a fresh liver tip was excised and cut into ~1 mm³ sections within 1–3 min, then immediately immersed in pre-chilled electron microscopy fixative and stored at 4 °C in the dark. A 2 × 2 × 2 mm liver sample was also collected from the same fish for ROS detection and was snap-frozen in liquid nitrogen. For transcriptomic analysis, liver tissues from these five fish were individually collected, and total RNA was extracted. All procedures were approved by the Animal Care Committee of Jiangsu Ocean University.

### 2.4 Total RNA extraction and real-time PCR analysis

To detect mRNA expression associated with hepatic inflammation and oxidative stress, total RNA was extracted from liver tissues with TRIzol reagent, after which chloroform extraction, isopropanol precipitation, and washing with 75% ethanol. RNA quality was then assessed on a 1.2% agarose denaturing gel. The absorbance of the RNA at 260/280 nm was measured using a UV-Vis spectrophotometer (Quawell Q5000, USA). Then cDNA was synthesized with HiScript II Q RT SuperMix (Vazyme, China), and RT-qPCR was performed on a Bioer FQD-96C system using ChamQ Blue Universal SYBR qPCR Master Mix (Vazyme, China). The thermal cycling protocol comprised an initial 30-s denaturation at 95°C, followed by 40 cycles at 95°C (10 s) and 60°C (30 s). Primers used in this study (**Table 1**), Relative transcript abundance was determined using the 2^(-ΔΔCT)^ method with β-actin as the endogenous reference (Livak and Schmittgen, 2001).

**Table 1:** Primer sequences.

Genes	F primer (5′to3′)	R primer (5′to3′)	Genbank no.	Product length
*gpx*	CCCTGATGACTGACCCAAAG	GCACAAGGCTGAGGAGTTTC	HS032063.1	174bp
*hsp47*	ACAAGGAGAACCGCATCTTCGTG	GCCAAGTGTCCACCTGCTTCC	XM_035625876.1	138bp
*hsp70*	CTGTCCCTGGGTATTGAGAC	GAACACCACGAGGAGCA	XM_047335714.1	220bp
*hsp90*	GTGAGACCACTTCCGCAGCCGT	ACGCAAGAGCAACACTGGTCCACC	XM_035606744.2	231bp
*ppar-γ*	AAGTGACGGAGTTCGCCAAGA	GTTCATCAGAGGTGCCATCA	XM_035631101.2	122bp
*stat1*	AGAGTGCCAACGAGAAAGCG	ACTGAACCGAAGCAGAAAGG	KJ156369.1	179bp
*stat2*	TGTCGGACCTAAATAAGCTCCT	CTCTGCCACTCCACTAGCTC	XM_035630581.2	82bp
*nrf-2*	CCACAACAACGCTCCCTTCACC	TTGGCCCTCTGCTCGTCTCTG	AWP13156.1	81bp
*sod*	AAACAATCTGCCAAACCTCTG	CAGGAGAACAGTAAAGCATGG	XM_035617519.2	165bp
*prdx6*	ACTGCCCGCTGTGTGTTTGTG	CGGCGTGGCAACCTTCTTCTG	ADJ57694.1	147bp
*tgf-β*	CTGCAGGACTGGCTCAAAGG	CATGGTCAGGATGTATGGTGGT	XM_035617102	186bp
*p38*	GAACGCCCCCAACATCTCTA	CTCGGCTGCTGTTATTCGC	SRP172919	185bp
*tnf-α*	GGGTGGATGTGGAAGGTGAT	GGCCTCTGTTTGGCTTGACT	XM_035638223.1	123bp
*mtor*	ACCGATCTGCCGTCATCATT	TGGAGCGGAAAAAGCCTTGA	XM_035644862	113bp
*il-1β*	GAGAGCATCGTGGAAGAACA	GTTTCGGACCAGAACGAAGT	XM_035640817.1	129bp
*jnk*	CTGGTAGAGCAGGTAGGACA	CACAGAAGACACTGGAAGAA	XM_035644965	122bp
*bcl-2*	TTATCAGCGGCATCTTCATCTC	TTGGCGAGGCGGTGTAATC	XM_020104180.1	113bp
*caspase-3*	TTCTGCCATTGTCTCTGTGC	GCCCTGCAACATAAAGCAAC	JU391554.1	130bp
*β-actin*	CGTGCGTGACATCAAGGAG	AGGAAGGAAGGCTGGAAGAG	EU686692.1	177bp

*gpx*, glutathione peroxidase; *hsp47*, *70*, *90*, heat shock proteins 47, 70, 90; *stat1*, *2*, signal transduction and transcription activators 1, 2; *ppar-γ*, Peroxisome proliferator-activated receptor γ; *nrf-2*, nuclear factor erythroid-2 related factor; *tnf-α*, tumor necrosis factor-α; sod, superoxide dismutase; *prdx-6*, peroxiredoxin-6; *tgf-β*, transforming growth factor-β; *p38*, p38 mitogen-activated protein kinase; *il-1β*, interleukin-1β; *jnk*, stress-activated protein kinase; *mtOR*, mammalian target of rapamycin; *bcl-2*, b-cell lymphoma-2; *caspase-3*, cysteinyl aspartate specific proteinase-3.

### 2.5 Preparation of liver transmission electron microscope sections

Hepatic sections were rinsed by 0.1 M PBS (pH 7.4), post-fixation was carried out with 1% osmium tetroxide at ambient temperature for 2 h protected from light, and then rinsed again. The tissue was sequentially dehydrated using a gradient of 30%, 50%, 70%, 80%, 95%, and two passes of 100% ethanol, each step was carried out for 20 min; after which two 15-min exchanges with absolute acetone were performed. The dehydrated tissue was permeabilized using a mixture of acetone and 812 embedding medium in varying ratios (1:1, permeabilized at 37°C for 2–4 h; 1:2, permeabilized overnight at 37°C), then transferred to pure 812 embedding medium and permeabilized at 37°C for 5–8 h. Tissue samples were embedded in a mold, left overnight at 37°C, and polymerized at 60°C for 2 days. After the polymerization process, the resin blocks were subjected to semi-thin sectioning and localized by toluidine blue staining, followed by ultrathin sectioning (60–80 nm) and sections were mounted on 150-mesh copper grids then stained with 2% uranyl acetate (ethanol, dark, 8 min), following rinsing, grids were stained with 2.6% lead citrate (8 min) with carbon dioxide excluded to prevent precipitate formation, followed by washing and drying overnight. At last, the sections were inspected and captured with a transmission electron microscope.

### 2.6 Preparation of liver ROS fluorescence staining sections

Liver samples were cut into sections at a thickness of approximately 10 μm using a freezing microtome. Frozen sections were thawed at room temperature, air-dried, and then the tissue regions were marked with a histochemical pen. A working solution was prepared by diluting the ROS fluorescent probe at a ratio of 1:500 in PBS, which was then applied to each tissue. The sections were then incubated in the dark at 37°C for 30 min. When incubation had finished, following washed the sections three times with PBS on a shaking platform, with each washed lasted 5 min. Excess liquid was gently shaken off, and then DAPI stain was applied; for nuclear counterstaining, sections were maintained in the dark under ambient conditions for 10 min. The sections were rinsed again with PBS three times (5 min each time). Finally, observation and image capture were performed under an upright fluorescence microscope using the following settings: DAPI was excited at 330–380 nm and detected at 420 nm, while ROS was excited at 520 nm and emission was collected at 605 nm.

### 2.7 Determination of oxidative stress biomarkers by ELISA

Oxidative stress was assessed by measuring 4-hydroxynonenal (4-HNE), 8-hydroxy-2’-deoxyguanosine (8-OHdG), and 8-iso-prostaglandin F2α (8-iso-PGF2α) levels with commercially available ELISA kits, per the manufacturer’s guidelines (Jingmei Biotechnology, China). Prior to each assay, samples were centrifuged at 3,000 rpm for 20 min at 2-8°C, and the supernatant fraction was harvested and kept for subsequent assays. Six-point standard curves were prepared using the provided calibrators. In each standard well, 50 μL of the corresponding standard solution was dispensed, whereas each sample well received 10 μL of sample and 40 μL of sample diluent, yielding a final 5-fold dilution. For the competitive ELISAs (4-HNE and 8-OHdG), 50 μL of HRP-conjugated competitive antigen was subsequently added to each well; for the sandwich ELISA (8-iso-PGF2α), 100 μL of HRP-conjugated detection antibody was used instead. Following sealing, ELISA plates were incubated for 60 min at 37°C, raised five times with wash buffer, then dispensed with 50 μL of Chromogen Solutions A and B, and incubated in the dark for 15 min at 37°C. After adding 50 μL of stop solution to halt the reactions then absorbance at 450 nm was measured within 15 min. For 4-HNE and 8-OHdG, the percent binding (B/B0) was calculated, and standard curves were constructed using logit-log transformation or four-parameter logistic curve fitting; sample concentrations were then interpolated and multiplied by the dilution factor of 5. For 8-iso-PGF2α, the standard curve was plotted as OD versus concentration, and sample values were read directly from the curve and similarly corrected for the 5-fold dilution. The assay sensitivities were 0.1 ng/mL, 1 ng/mL, and 1 pg/mL for 4-HNE, 8-OHdG, and 8-iso-PGF2α, respectively.

### 2.8 Liver transcriptomic analysis

#### 2.8.1 Database construction, quality control, and sorting

For every group, four mixed liver samples were randomly collected for total RNA extraction. Subsequently, Novogene Co., Ltd. (China) performed library preparation, quality control, sequencing, and bioinformatics analysis. Enrichment of mRNA was performed using magnetic beads, and fragmentation was performed in a fragmentation buffer containing divalent cations. RNA was degraded using RNaseH and used together with the fragmented mRNA for cDNA synthesis. After reverse transcription, the purified cDNA was ligated to adapters and amplified by PCR. The resulting amplicons were subsequently purified with AMPure XP beads for sequencing library preparation. Afterward, the fragment size distribution was evaluated with an Agilent 2100 Bioanalyzer to confirm library integrity. After library verification, paired-end sequencing (2 × 150 bp) was performed on the Illumina NovaSeq 6000 system.

#### 2.8.2 Bioinformatics analysis

Raw image files from the sequencer were processed with consensus assessment of sequence and variation (CASAVA) for base calling and conversion into raw reads. Reads containing adapters, poly-N sequences, and low-quality reads were then filtered out. At the same time, quality metrics (Q20, Q30, GC content) for the clean data were determined using fastp software (version 0.19.7). Further analyses were conducted on the basis of clean data to maintain high analytical standards. In this study, a pre-indexed reference genome was generated using HISAT2 (version 2.0.5), and paired-end clean reads were then mapped to the reference transcriptome.

New gene predictions were made using StringTie (1.3.3b). Next, read counts per gene were determined using featureCounts (version 1.5.0-p3). The FPKM of each gene was computed according to its sequence length and mapped read count. Subsequently, DEGs between the two groups were classified using DESeq2 (version 1.20.0), with the following parameters: adjusted P-value (Padj) ≤ 0.05 and |log_2_FoldChange| ≥ 1.0. The clusterProfiler package (version 3.8.1) was used to perform GO and KEGG enrichment analyses on the DEGs. Co-expression network analysis was conducted with the WGCNA R package (version 3.5.0), which provides a set of functions for calculating various weighted correlation analyses. The gene network node relationships from the correlation module were imported into Cytoscape (version 3.0; Cytoscape Consortium, USA) for visualization and editing, and hub genes were identified. The raw data of this study have been deposited in the NCBI database with the BioProject accession PRJNA1457694 and are accessible in the SRA.

### 2.9 Data analysis

Statistical analysis was performed using IBM SPSS Statistics 22.0 (IBM Corp., USA). Data are expressed as mean ± SD. Group differences were analyzed by one-way ANOVA with Tukey’s *post hoc* test. Statistical significance was set at *P* < 0.05. The bar charts were generated using GraphPad Prism 9 (GraphPad Software, USA).

## 3 Results

### 3.1 Transmission electron microscopic observations of the liver

**Figure 1:**
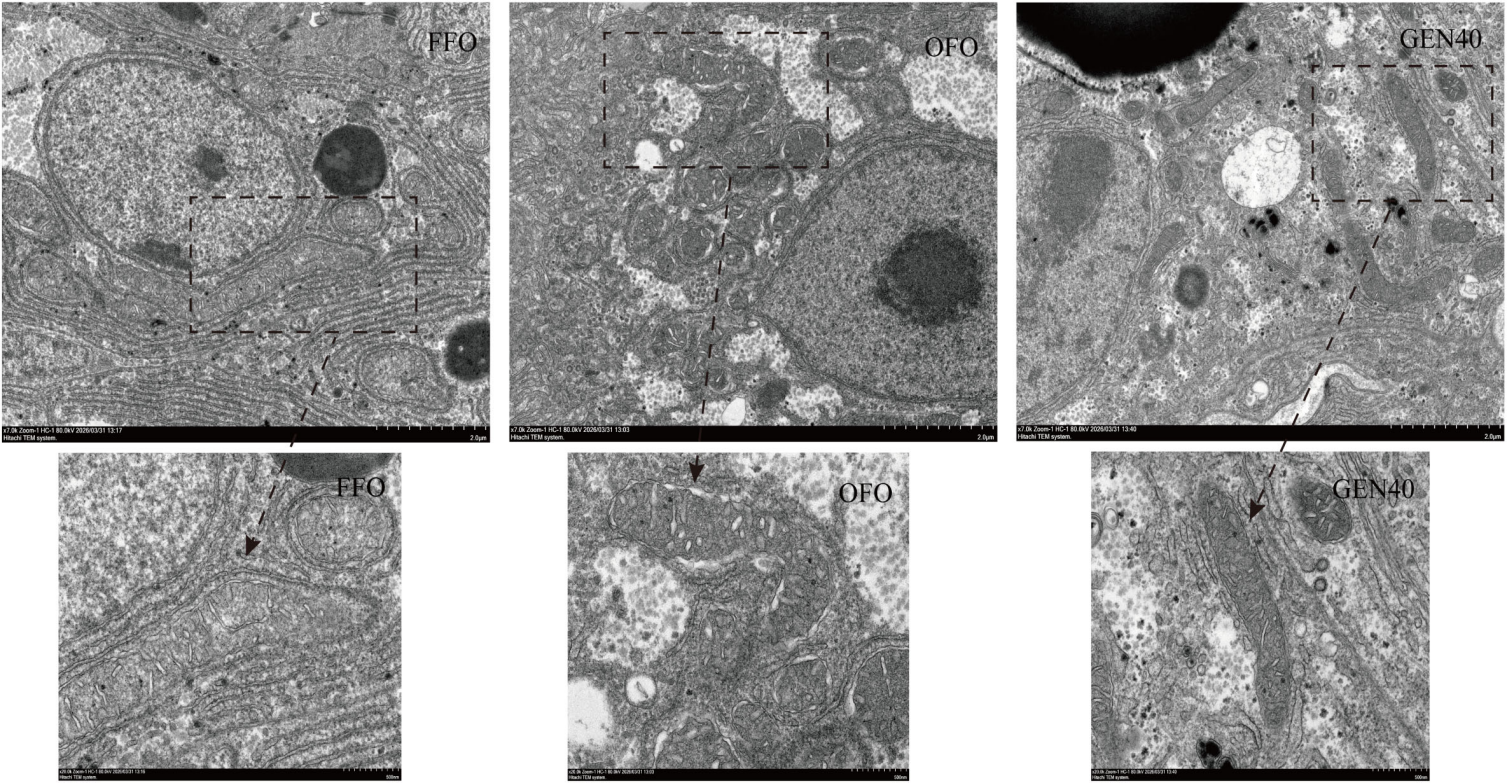
Transmission electron microscopy (TEM) observation of the ultrastructure of mitochondria in the livers of turbot from different groups. Scale bar = 2 μm, 500nm.

Liver mitochondrial ultrastructure was assessed by transmission electron microscopy in turbot from each experimental group (**Figure 1**). In the FFO group, the mitochondria in liver cells exhibited regular morphology, with clear and intact cristae and continuous membrane structures. In the OFO group, mitochondria exhibited significant damage: swelling, cristae rupture, and vacuolization, with loss of membrane integrity of the outer mitochondrial membrane. Compared to the OFO group, mitochondrial damage was markedly decreased in the GEN40 group, with reduced swelling, partial restoration of cristae structure, and reduced vacuolization.

### 3.2 Measurement of ROS fluorescence intensity in the liver

**Figure 2:**
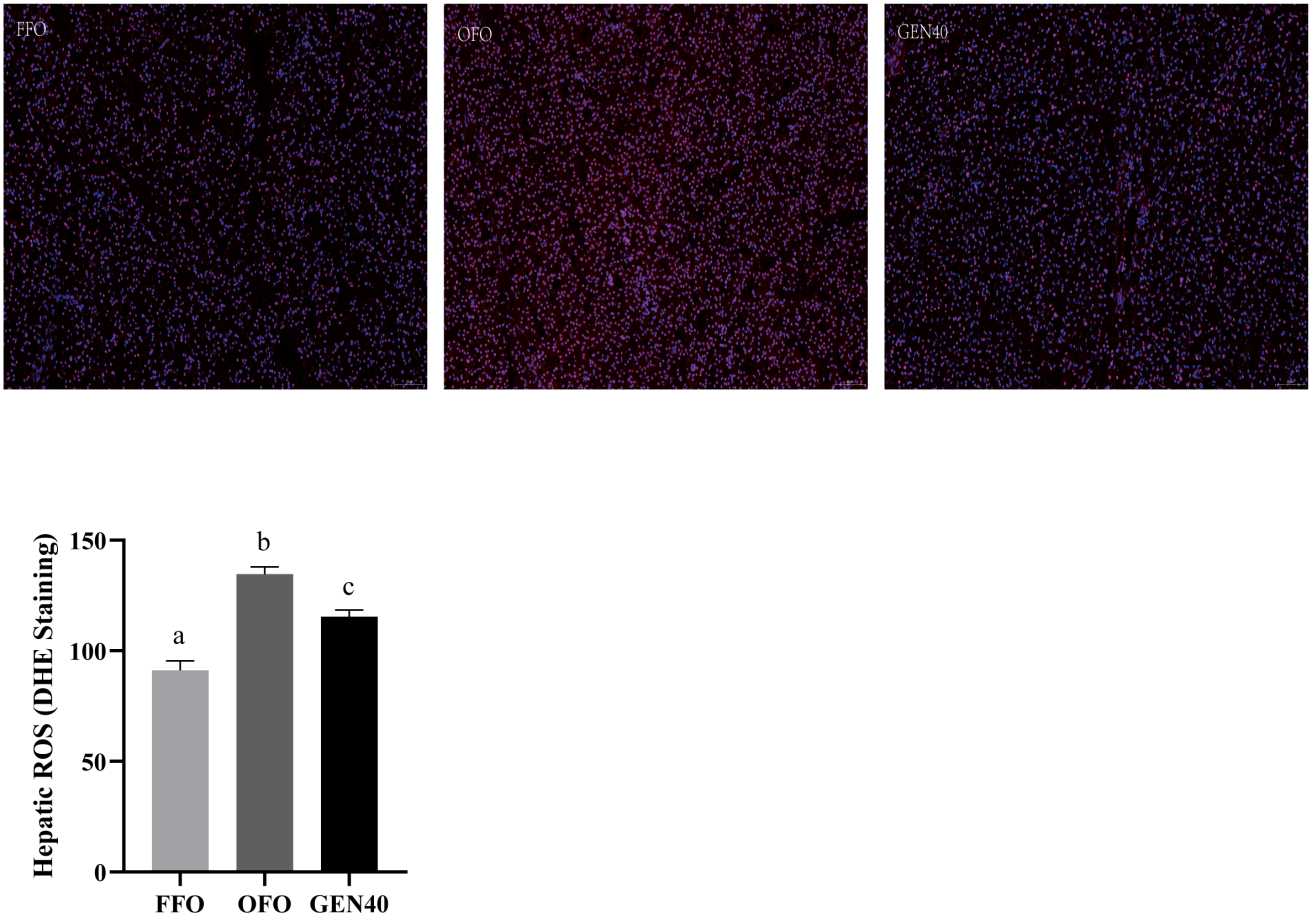
Detection of ROS levels in liver tissues by DHE staining, ros staining images of liver tissue from each group (scale bar=50 μm). Semi-quantitative statistical analysis of the mean fluorescence intensity across groups was performed; data are presented as Mean ± SD (n=5). Different letters indicate significant differences among the FFO, OFO, and GEN40 groups (one-way ANOVA followed by Tukey’s multiple comparison test, *P* <0.05).

Hepatic oxidative stress levels were assessed using dihydroethidium (DHE) fluorescence staining. The findings revealed that, compared with the FFO and GEN40 groups, the red fluorescence in liver tissue from the OFO group was stronger; the red fluorescence intensity in the GEN40 group was elevated in comparison with that in the FFO group but decreased relative to that in the OFO group. Subsequent statistical analysis of the mean fluorescence intensity of DHE staining (**Figure 2**) revealed that the mean fluorescence intensity in liver tissue was significantly higher in the OFO group compared with FFO and GEN40 groups, indicating a substantial rise in superoxide anion levels in the OFO group (*P* < 0.05); in contrast, the superoxide anion concentration in the GEN40 group was markedly reduced compared to the OFO group (*P* < 0.05).

### 3.3 Gene expression

#### 3.3.1 Expression of genes associated with apoptosis

**Figure 3:**
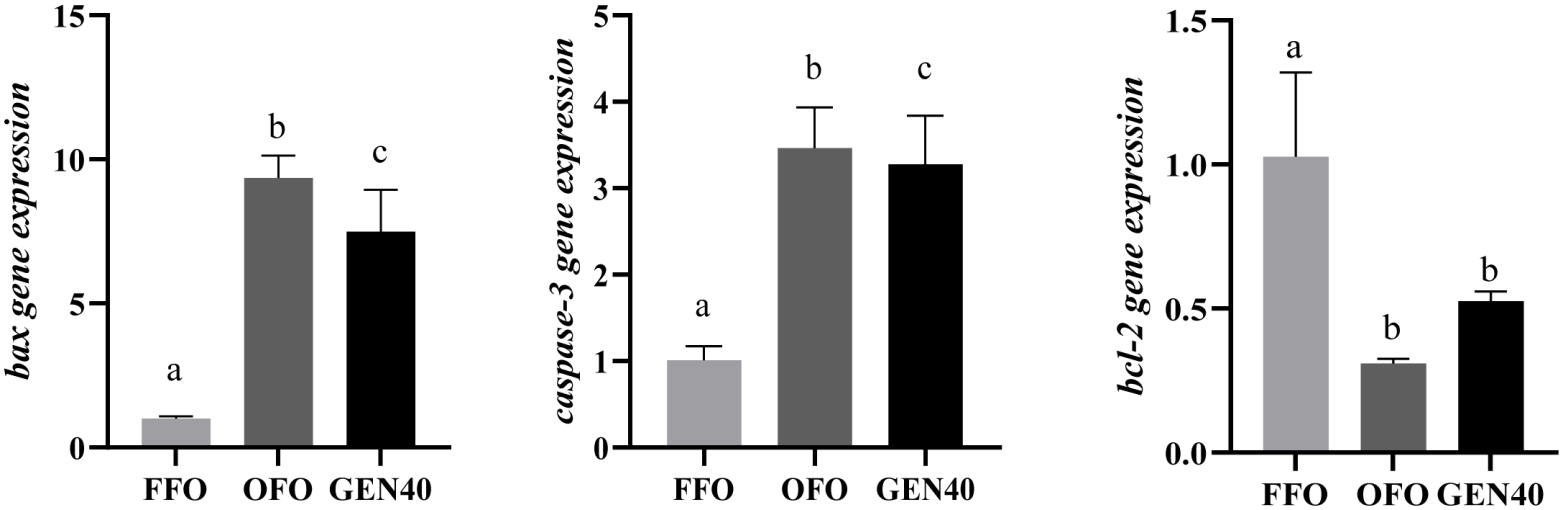
Apoptosis-related gene expression in turbot liver after genistein exposure. Data are presented as Mean ± SD (n=6). Different letters indicate significant differences among the FFO, OFO, and GEN40 groups (one-way ANOVA followed by Tukey’s multiple comparison test, *P* < 0.05). *bax*, bcl2-associated X protein; *caspase-3*, Cysteinyl Aspartate Specific Proteinase-3; *bcl-2*, B-cell lymphoma-2.

The mRNA level of bcl2-associated X protein (*bax*) in turbot from the OFO group was significantly upregulated relative to the FFO group (*P* < 0.05); the relative mRNA level of *bax* in turbot from the GEN40 group was remarkably reduced relative to the OFO group (*P* < 0.05); and the relative expression of *bax* mRNA in turbot from the GEN40 group was markedly in excess of that in the FFO group (*P* < 0.05). At the same time, the transcriptional level of cysteinyl aspartate specific proteinase-3 (*caspase-3*) mRNA in the OFO group was pronouncedly increased in comparison with the GEN40 group (*P* < 0.05); the transcript abundance of *caspase-3* in the GEN40 group was notably lower than that in the OFO group (*P* < 0.05); and the relative expression level of *caspase-3* mRNA in the GEN40 group was pronouncedly elevated compared with that in the FFO group (*P* < 0.05). The relative transcript level of b-cell lymphoma-2 (*bcl-2*) in the FFO group was considerably increased compared with the OFO and GEN40 groups (*P* < 0.05), and the transcript level of *bcl-2* mRNA in the GEN40 group was upregulated versus the OFO group (*P* < 0.05) (**Figure 3**).

#### 3.3.2 Expression of oxidative stress-related genes in the liver

**Figure 4:**
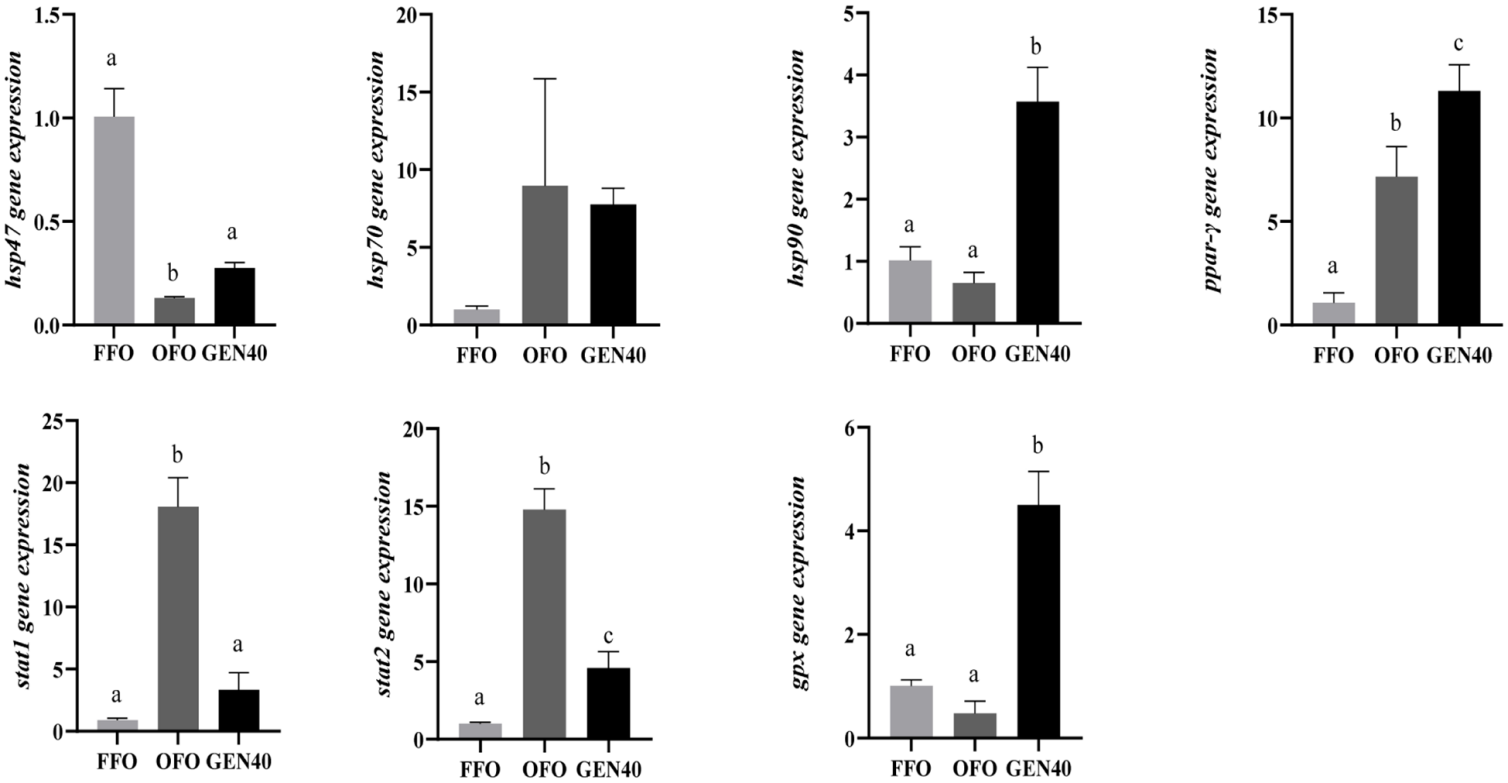
The influence of dietary genistein on liver oxidative stress gene transcription in turbot. Data are presented as Mean ± SD (n=6). Different letters indicate significant differences among the FFO, OFO, and GEN40 groups (one-way ANOVA with Tukey’s *post hoc* test, *P* < 0.05). *hsp47*, *70*, *90*: heat shock proteins 47, 70, 90; *gpx*, glutathione peroxidase; *ppar-γ*, Peroxisome proliferator-activated receptor γ; *stat1, 2*, signal transduction and transcription activators 1, 2.

The quantified expression of heat shock protein 47 (*hsp47*) mRNA in turbot from the FFO group was notably upregulated versus the OFO group (*P* < 0.05), and the mRNA level of *hsp47* in turbot from the GEN40 group was considerably higher than that in the OFO group (*P* < 0.05), whereas the relative expression level of *hsp47* mRNA didn’t differ markedly between the FFO and GEN40 groups (*P*>0.05). No marked variations in the relative level of heat shock protein 70 (*hsp70*) mRNA in turbot among the FFO, OFO, and GEN40 groups (*P*>0.05). The heat shock protein 90 (*hsp90*) mRNA abundance in turbot from the GEN40 group showed a significant increase relative to that in the FFO and OFO groups (*P* < 0.05), while there were no appreciable disparities in the relative transcript abundance of *hsp90* mRNA between the FFO and OFO groups (*P*>0.05). The relative of *gpx* mRNA in the GEN40 group of turbot was markedly higher than that in the FFO and OFO groups (*P* < 0.05), while no statistically significant difference in *gpx* mRNA level of between the FFO and OFO groups (*P*>0.05). The relative mRNA level of peroxisome proliferator-activated receptor γ (*ppar-γ*) in the GEN40 group of turbot was substantially increased in that in the FFO and OFO groups (*P* < 0.05), and the relative expression of *ppar-γ* mRNA in the OFO group was also significantly upregulated in the FFO group (*P* < 0.05). The expression of signal transducer and activator of transcription 1 (*stat1*) mRNA in the OFO group was markedly higher than that in the FFO and GEN40 groups (*P* < 0.05), while the relative mRNA levels of *stat1* did not differ significantly between the FFO and GEN40 groups (*P*>0.05). The relative mRNA levels of signal transducer and activator of transcription 2 (*stat2*) in turbot were markedly higher than that in the OFO group relative to the FFO and GEN40 groups (*P* < 0.05), and the expression of turbot *stat2* mRNA in the GEN40 group was also markedly elevated in comparison to that in the FFO group (*P* < 0.05) (**Figure 4**).

#### 3.3.3 Expression of inflammation-related genes in the liver

**Figure 5:**
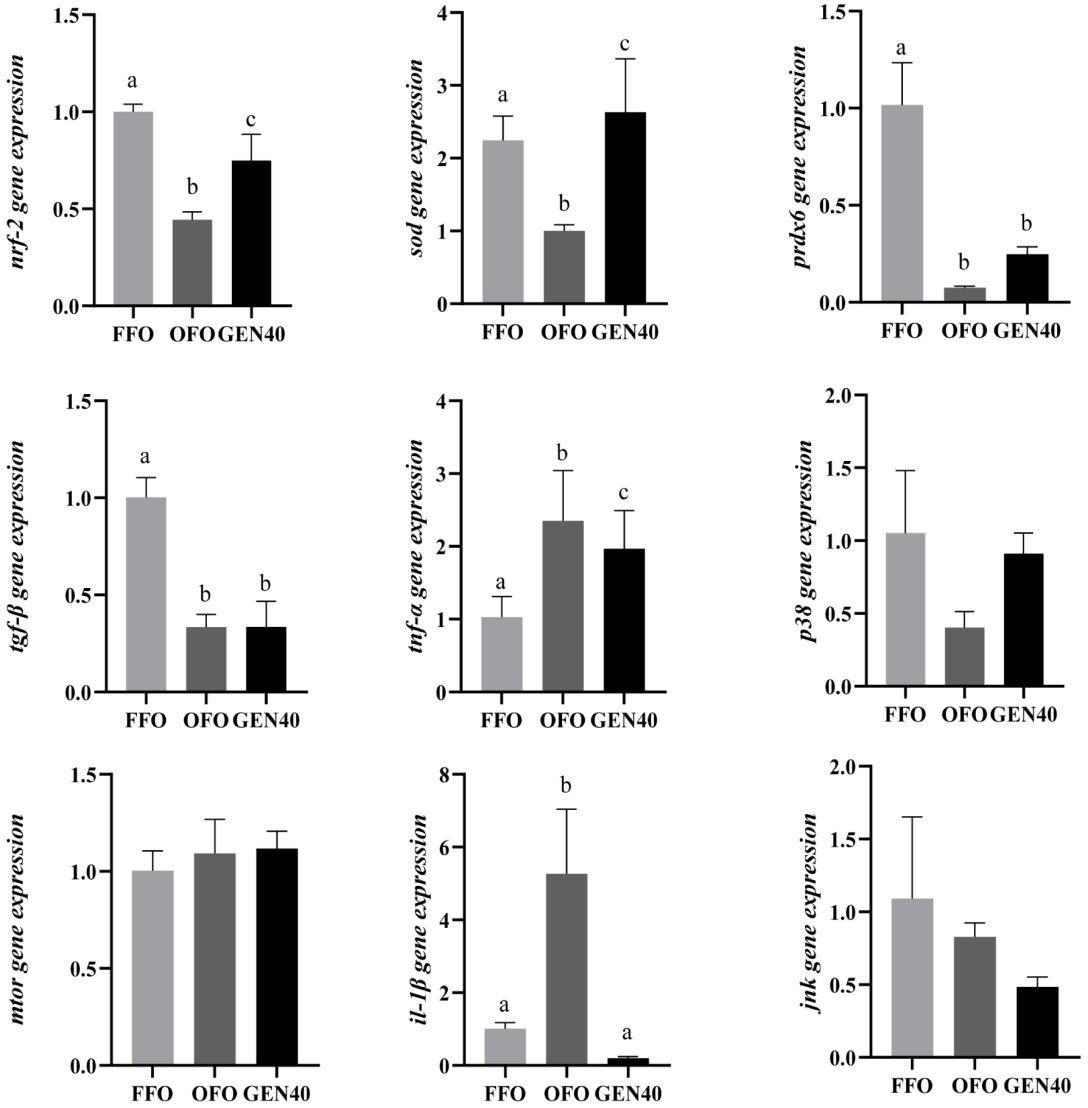
Modulation of inflammatory cytokine gene transcription by dietary genistein in the intestine of turbot data are presented as Mean ± SD (n=6). Different letters indicate significant differences among the FFO, OFO, and GEN40 groups (one-way ANOVA with Tukey’s *post hoc* test, *P* < 0.05). *nrf-2*, nuclear factor erythroid-2 related factor; *sod*, superoxide dismutase; *prdx-6*, peroxiredoxin-6; *tgf-β*, transforming growth factor-β; *p38*, p38 mitogen-activated protein kinase; *tnf-α*, tumor necrosis factor-α; *mTOR*, mammalian target of rapamycin; *il-1β*, interleukin-1β; *jnk*, c-Jun N-terminal Kinase.

The relative mRNA level of turbot nuclear factor erythroid 2-related factor 2 (*nrf-2*) was markedly higher in the FFO group compared with the OFO and GEN40 groups (*P* < 0.05), and the relative expression of *nrf-2* mRNA in turbot from the OFO group was notably reduced compared to that in the GEN40 group (*P* < 0.05). The relative expression of *sod* mRNA in turbot from the OFO group was considerably reduced compared to that in the FFO and GEN40 group (*P* < 0.05), and the relative expression of *sod* in the GEN40 group was significantly higher than that in the FFO coup (*P* < 0.05). The expression level of Peroxiredoxin 6 (*prrdx6*) mRNA in the FFO group of turbot was significantly higher than that in the OFO and GEN40 groups (*P* < 0.05), while there showed comparable levels of *prrdx6* mRNA across the OFO and GEN40 groups (*P*>0.05). The expression level of transforming growth factor-β (*tgf-β*) mRNA in turbot from the FFO group was markedly upregulated relative to that in the OFO and GEN40 groups (*P* < 0.05), while no notable variation was observed within the OFO and GEN40 groups (*P*>0.05). There was no significant difference in p38 mitogen-activated protein kinase (*p38*) mRNA level in turbot among the FFO, OFO, and GEN40 groups (*P*>0.05). The normalized mRNA level of tumor necrosis factor-α (*tnf-α*) in turbot were markedly higher in the OFO group relative to that in the FFO and GEN40 groups (*P* < 0.05), and the expression of *tnf-α* was noticeably increased in comparison with that in the FFO group (*P* < 0.05). Mammalian target of rapamycin (*mtor*) mRNA level was similar among the FFO, OFO, and GEN40 groups (*P*>0.05). In the OFO group, expression of Interleukin-1β (*il-1β*) mRNA was notably up-regulated as opposed to that in the FFO and GEN40 groups (*P* < 0.05); however, *il-1β* mRNA level showed no notable variation across the FFO and GEN40 groups (*P*>0.05). The mRNA expression of c-Jun N-terminal Kinase (*JNK*) didn’t differ significantly among the FFO, OFO, and GEN40 groups (*P*>0.05) (**Figure 5**).

### 3.4 Measurement of serum oxidative stress markers

**Figure 6:**
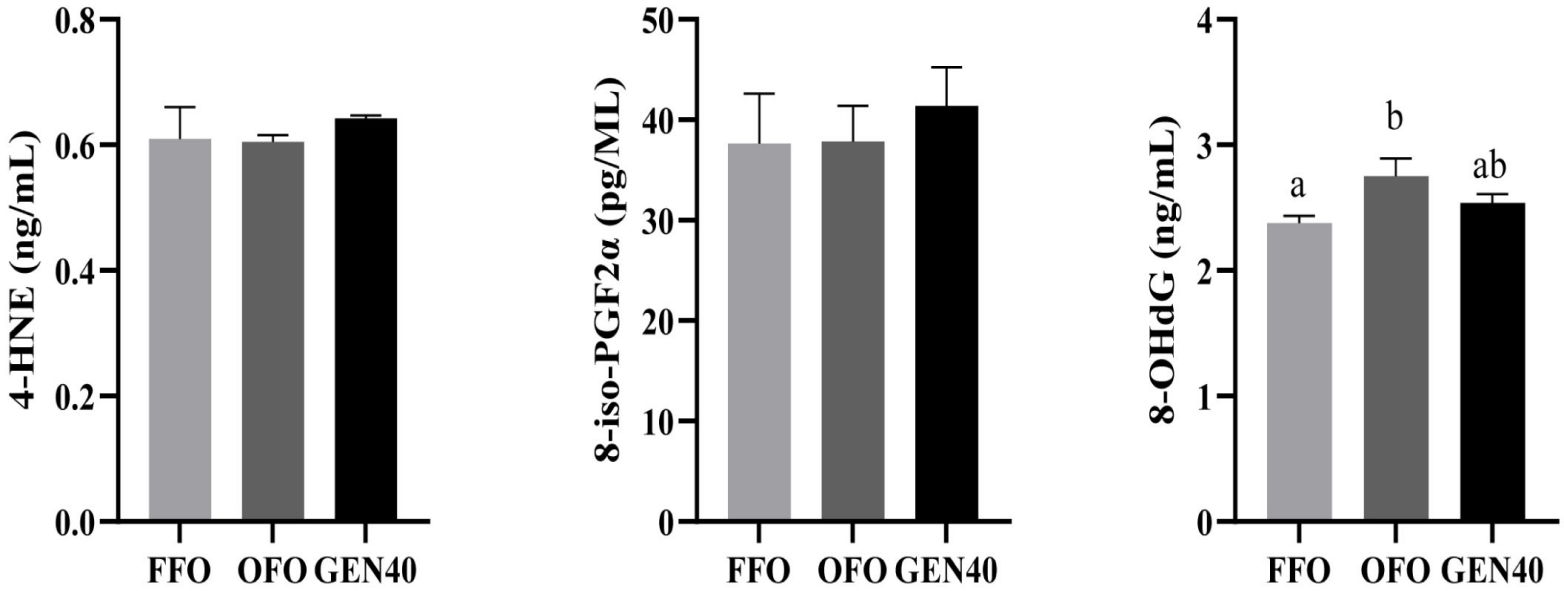
Effects of genistein on serum antioxidant indices in turbot. Data are presented as Mean ± SD (n=6). Different letters indicate significant differences among the FFO, OFO, and GEN40 groups (one-way ANOVA with Tukey’s *post hoc* test, *P* < 0.05). 8-iso-PGF2α, 8-iso-prostaglandin F2α; 4-HNE, 4-hydroxy-2-nonenal; 8-OHdG, 8-hydroxy-2’-deoxyguanosine.

The oxidative stress biomarkers 4-HNE and 8-iso-PGF2α showed no statistically substantial distinction in their concentrations among the FFO, OFO, and GEN40 groups (*P*>0.05), at same time the oxidative stress biomarkers 8-OHdG concentration in the OFO group was markedly higher than that in the FFO group (*P* < 0.05), whereas the GEN40 group showed an intermediate level that was not markedly distinguished from either the FFO or the OFO group (*P*>0.05) (**Figure 6**).

### 3.5 Transcriptome analysis of the liver

Consistent with earlier reports, it was found that adding genistein to fish oil-based feed significantly alleviated oxidative injury and inflammatory reactions in the livers of turbot. To further investigate the critical signal transduction pathways and hub genes involved in GEN-mediated mitigation of oxidative damage and inflammatory responses in turbot livers, liver transcriptomic analyses were conducted on the FFO, OFO, and GEN40-treated groups, with a primary focus on how these pathological processes affect biological functions.

#### 3.5.1 Assessment of sequencing data quality

RNA-Seq statistical data (**Supplementary Materials**) showed that, a total of 730,521,912 raw sequencing reads were obtained. Once the data were filtered and the sequencing error rates and GC distribution were examined, 105.26 G of valid bases were obtained, resulting in 718,386,780 valid sequences, the GC content was essentially consistent across samples, the sequencing error rate was 0.01%, and the Q20 and Q30 values exceeded 98% and 95%, while indicating that RNA-seq were of sufficient integrity for all subsequent analyses. Box-and-whisker plots were generated to illustrate and compare the expression level distributions across the different samples. As shown, gene expression values are generally highly similar across the groups, although there were minor differences between them (**Supplementary Materials**).

#### 3.5.2 Differential gene analysis

Analysis of the differential gene Venn diagram (**Supplementary Materials**) revealed that there were 1,526 DEGs between the FFO group and the OFO group, 887 DEGs across the FFO group and the GEN40 group, and 1,373 DEGs across the GEN40 group and the OFO group.

Relative to the FFO group, the OFO group had 2,886 DEGs in the liver, including 1,428 overexpressed genes and 1,458 suppressed genes; compared to the FFO and OFO groups, the GEN40 group revealed 1,852 and 2,953 DEGs in the liver, respectively, of which 1,241 and 1,680 were upregulated, and 611 and 1,273 were downregulated. Afterward, the identified DEGs were analyzed. A comparison of the volcano plot and the clustering heatmap (**Supplementary Materials**) revealed good biological reproducibility among the sample groups and significant differences in gene expression levels.

Furthermore, transcriptomic analysis revealed coordinated changes in gene expression that were associated with oxidative stress and inflammation. Upregulated genes, including potassium inwardly rectifying channel subfamily J member 15 (*kcnj15*), transient receptor potential cation channel subfamily M member 5 (*trpm5*), glucagon b (*gcgb*), phosphofructokinase, muscle a (*pfkma*), and epithelial mitogen (*epgn*), were linked to calcium signaling, metabolic reprogramming, and tissue repair (**Table 2**). In contrast, downregulated genes such as arginase 2 (*arg2*), phosphoserine aminotransferase 1 (*psat1*), fatty acid synthase (*fasn*), insulin induced gene 1 (*insig1*), and stearoyl-CoA desaturase (*scd*) were mostly involved in metabolic pathways, and their suppression was thought to reflect a reduced anabolic capacity under stress conditions (**Table 3**). Cell cycle- and immune-related genes were also affected: kinesin family member 11 (*kif11*) and sperm antigen with calponin homology and coiled-coil domains 1 (specc1) were upregulated, whereas cyclin-dependent kinase inhibitor 1D (*cdkn1d*), syntaxin 7-like (*stx7l*), and mfhas1 were downregulated, suggesting increased proliferative activity alongside impaired vesicular trafficking and innate immune function. In addition, the downregulation of clock circadian regulator a (*clocka*) pointed to a disruption in circadian rhythm, which has been closely linked to redox homeostasis.

**Table 2:** Significantly upregulated genes from transcriptome analysis.

Gene description	Log2F C ^1^	P value ^2^
Sperm antigen with calponin homology and coiled-coil domains 1	1.89503271838526	2.10E-05
Kinesin family member 11	2.1935862458958	2.11E-05
Potassium inwardly rectifying channel subfamily J member 15	2.63911829211655	2.55E-05
Neurofascin homolog a	3.58734394572204	3.85E-05
Glucagon b	4.34474024521779	3.78E-18
Phosphofructokinase, muscle a	6.98900495039155	1.84E-06
Transient receptor potential cation channel, subfamily M, member 5	1.95579136079826	3.28E-06
Annexin A14	2.45216580490603	3.73E-06
Actin alpha 1, skeletal muscle b	9.41925081917398	1.57E-05
Epithelial mitogen	6.3023184572849	1.88E-05

^1^log2FC (s1/s2): the base-2 logarithm of the fold change of the gene/transcript between the two samples.

^2^P value: the significance test result of the difference between the two samples of the gene/transcript.

**Table 3:** Significantly downregulated genes from transcriptome analysis.

Gene description	Log2F C ^1^	Pvalue ^2^
Arginase 2	-1.85233746659118	4.21E-07
Cyclin-dependent kinase inhibitor 1D	-1.01811537965364	0.0002797
Phosphoserine aminotransferase 1	-2.8219797148842	0.0005276
Fatty acid synthase	-1.62916692001076	0.0005416
Insulin induced gene 1	-1.22158675305388	0.0007404
Clock circadian regulator a	-1.70721444861836	0.0007459
Syntaxin 7-like	-0.687449648841141	0.0011200
Stearoyl-CoA desaturase (delta-9-desaturase)	-2.41814848632162	0.0011439
Multifunctional ROCO family signaling regulator 1	-0.770816569502383	0.0012362
Glutaryl-CoA dehydrogenase a	-1.22395145621559	0.0013020

^1^log2FC (s1/s2): the base-2 logarithm of the fold change of the gene/transcript between the two samples;.

^2^P value: the significance test result of the difference between the two samples of the gene/transcript.

#### 3.5.3 Functional and pathway enrichment of DEGs

**Figure 7:**
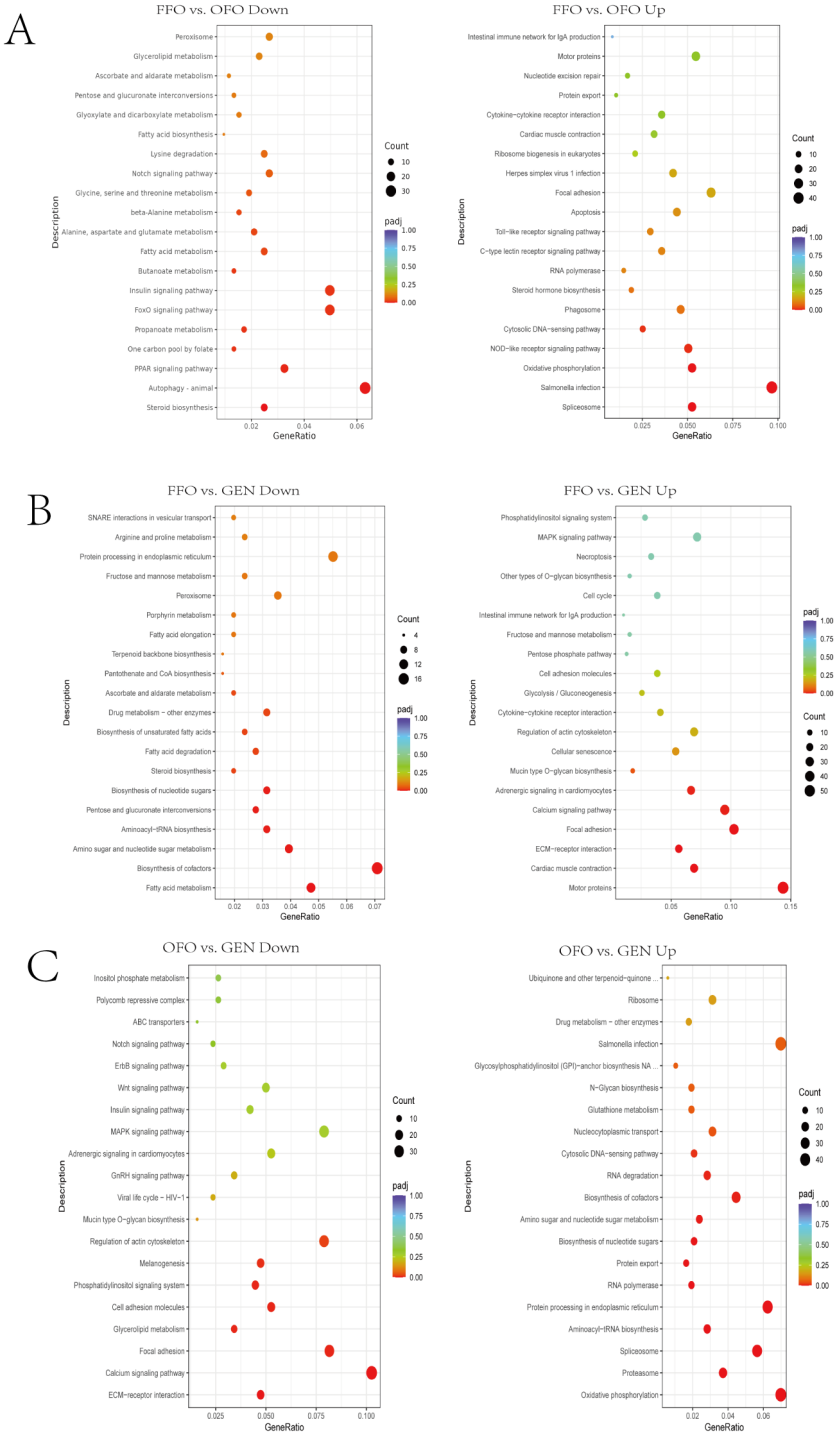
KEGG enrichment analysis of differentially expressed genes (DEGs) among the FFO, OFO, and GEN40 groups. **(A)** FFO vs OFO; **(B)** FFO vs GEN40; **(C)** OFO vs GEN40 (n=5).

Using KEGG analysis, hepatic DEGs were identified and analyzed in FFO groups and OFO groups. The outcomes revealed that, compared to FFO groups (**Figure 7A**), the genes significantly downregulated in the OFO group were primarily enriched in pathways associated with oxidative damage, including autophagy, PPAR signaling, fatty acid and glycine/serine/threonine metabolism, and the peroxisomal pathway (*P* < 0.05). Meanwhile, in contrast to FFO groups, the genes significantly upregulated in OFO groups were primarily concentrated in signaling pathways related to metabolic regulation and immune defense. These signaling pathways included the intestinal immune network for IgA, cytokine-cytokine receptor interactions, Toll-like and NOD-like receptor signaling, Salmonella infection response, oxidative phosphorylation, and ribosome biogenesis in eukaryotes (*P* < 0.05).

In comparison with FFO groups (**Figure 7B**), the downregulated DEGs in GEN groups were primarily enriched in signaling pathways related to basic metabolism and synthetic functions, including vesicular transport (SNARE interactions), ER protein processing, fatty acid metabolism (degradation, elongation, unsaturated biosynthesis), cofactor and aminoacyl-tRNA biosynthesis, peroxisomes, and arginine/proline metabolism (*P* < 0.05). Relative to FFO groups, the differentially upregulated genes in the GEN40 group were primarily enriched in signaling pathways related to immune activation, inflammation, and cell proliferation, including the phosphatidylinositol signaling system, the intestinal immune network for IgA, cytokine-cytokine receptor interactions, necroptosis, cell cycle and actin cytoskeleton regulation, and ECM-receptor interaction (*P* < 0.05).

In contrast to OFO groups, the downregulated DEGs in GEN40 groups were mainly enriched in signaling pathways connected to oxidative stress and inflammation (**Figure 7C**). These included the calcium signaling pathway, adrenergic signaling in cardiomyocytes, actin cytoskeleton and focal adhesion regulation (*P* < 0.05). Concurrently, the upregulated DEGs were markedly accumulated in signaling pathways related to biosynthesis and energy metabolism, including oxidative phosphorylation, Salmonella infection, biosynthesis of cofactors, ribosome, aminoacyl-tRNA synthesis, proteasome/endoplasmic reticulum processing, spliceosome, RNA degradation, nucleocytoplasmic transport (*P* < 0.05).

**Figure 8:**
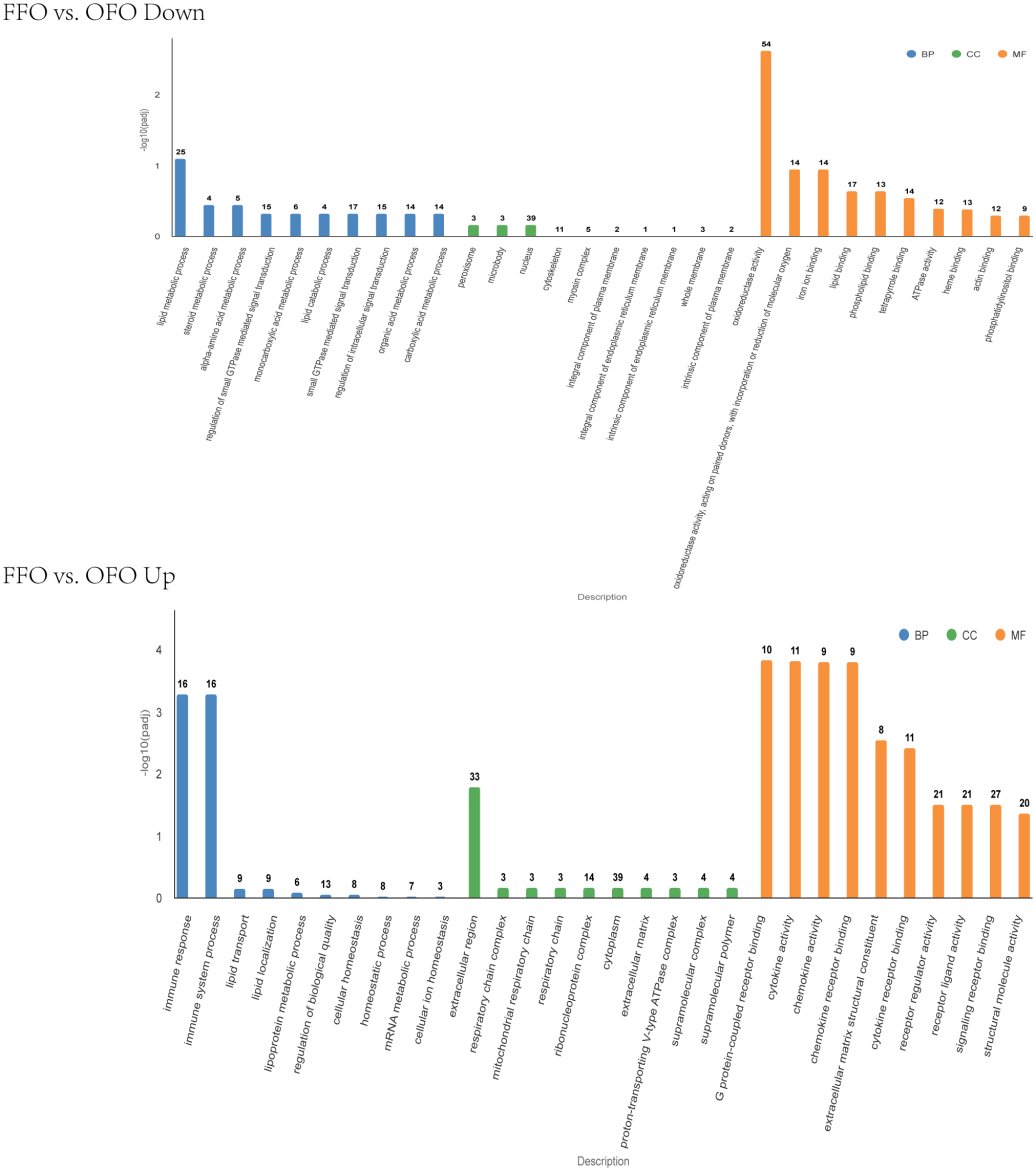
GO analysis of DEGs across FFO and OFO groups BP, Biological Process; CC, Cellular Component; MF, Molecular Function (n=5).

GO enrichment analysis results showed that compared with FFO groups (**Figure 8**), genes significantly downregulated in OFO groups were primarily enriched in the biological process (BP) category in areas such as lipid catabolism and small GTPase-mediated signal transduction regulation; in the cellular component (CC) category, they exhibited marked overrepresentation in terms related to the cell nucleus. Molecular function (MF) analysis results indicated that the relevant genes were primarily participated in processes such as redox reactions, iron ion binding, and lipid binding. In the BP classification, significantly upregulated genes were primarily enriched in terms such as immune response. In the CC classification, they were primarily localized to the extracellular space and cytoplasm. The MF analysis results indicated that the relevant genes were primarily involved in processes such as G protein-coupled receptor (GPCR) binding, cytokine activity, and receptor regulation.

GPCR binding, cytokine activity, and receptor regulatory activity.

**Figure 9:**
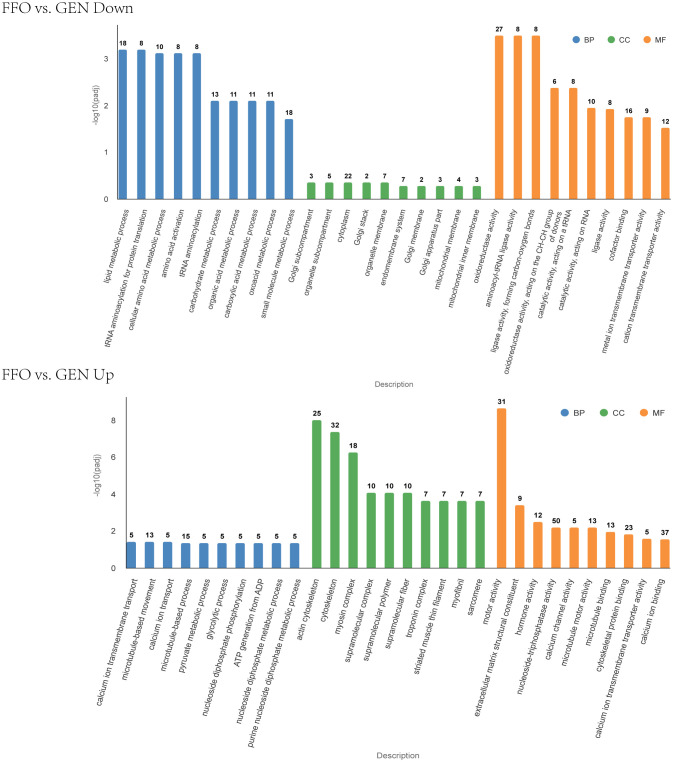
GO analysis of DEGs across FFO and GEN40 groups BP, Biological Process; CC, Cellular Component; MF, Molecular Function (n=5).

In comparison with FFO groups (**Figure 9**), it was also found that the downregulated DEGs in GEN40 groups were primarily within lipid metabolism within the BP category, and were mainly localized to organelle subcompartments and organelle membranes within the CC category. Analysis of MF indicated that the relevant genes were primarily involved in oxidoreductase activity and cofactor binding. Significantly upregulated genes were primarily enriched in the transport of substances and nucleoside diphosphate metabolic process within the BP classification, and were chiefly localized to the cytoskeleton and other areas within the CC classification. The MF analysis indicated that the relevant genes were primarily involved in structural molecule activity and nucleoside triphosphatase activity.

**Figure 10:**
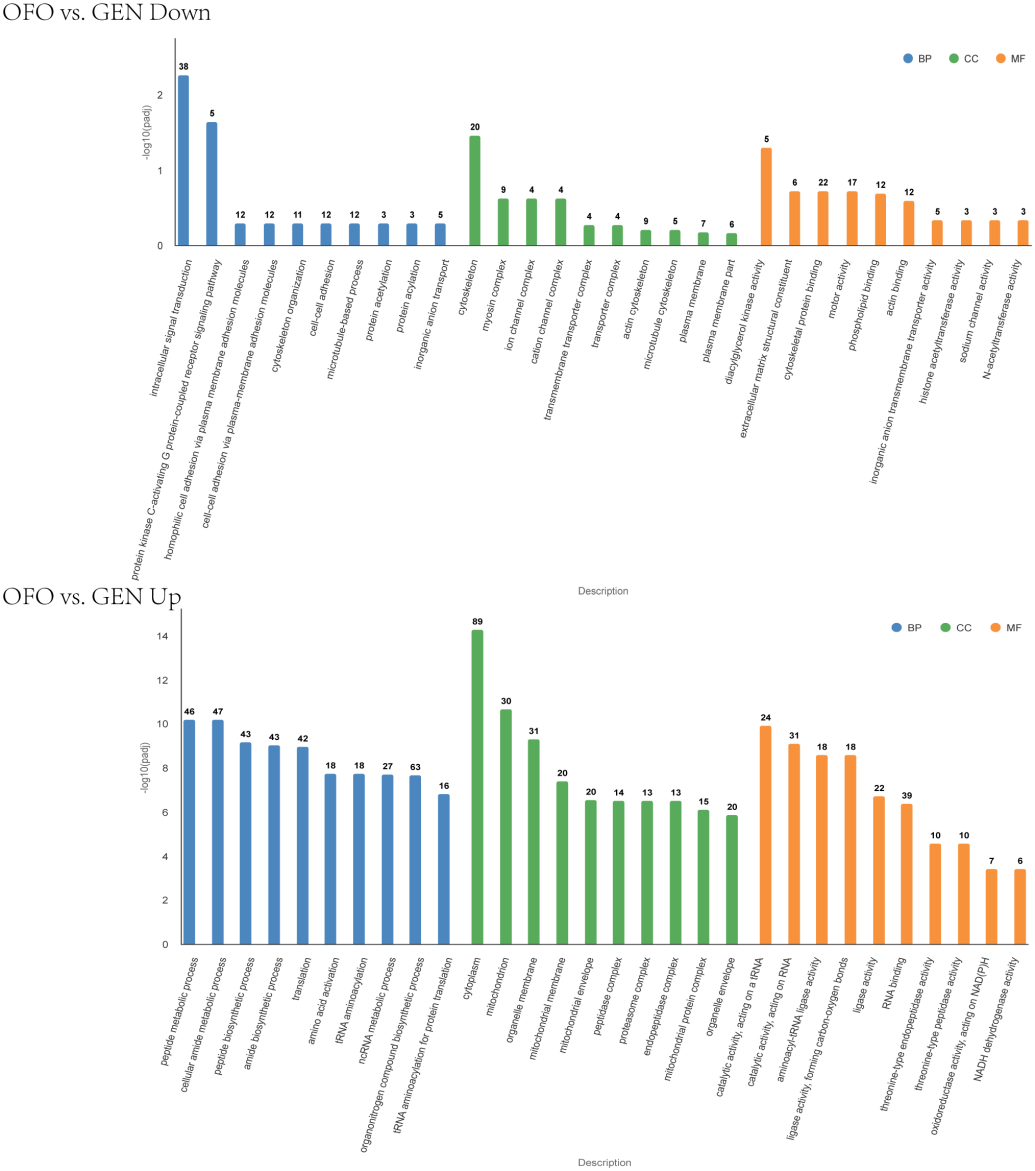
GO analysis of DEGs across OFO and GEN40 group BP, Biological Process; CC, Cellular Component; MF, Molecular Function (n=5).

Meanwhile, relative to OFO groups, the downregulated DEGs in GEN40 groups were primarily enriched in intracellular signal transduction within the BP category, and were mainly localized to the cytoskeleton and myosin complexes within the CC category. Analysis of MF indicated that these genes were primarily involved in diacylglycerol kinase activity. Significantly upregulated genes were primarily enriched in the BP category for peptide metabolism and cellular amide metabolism, and in the CC category for the cytoplasm. The MF analysis results indicated that the relevant genes were primarily involved in catalytic activity, specifically tRNA-binding catalytic activity (**Figure 10**).

#### 3.5.4 Hub genes exploration by WGCNA

**Figure 11:**
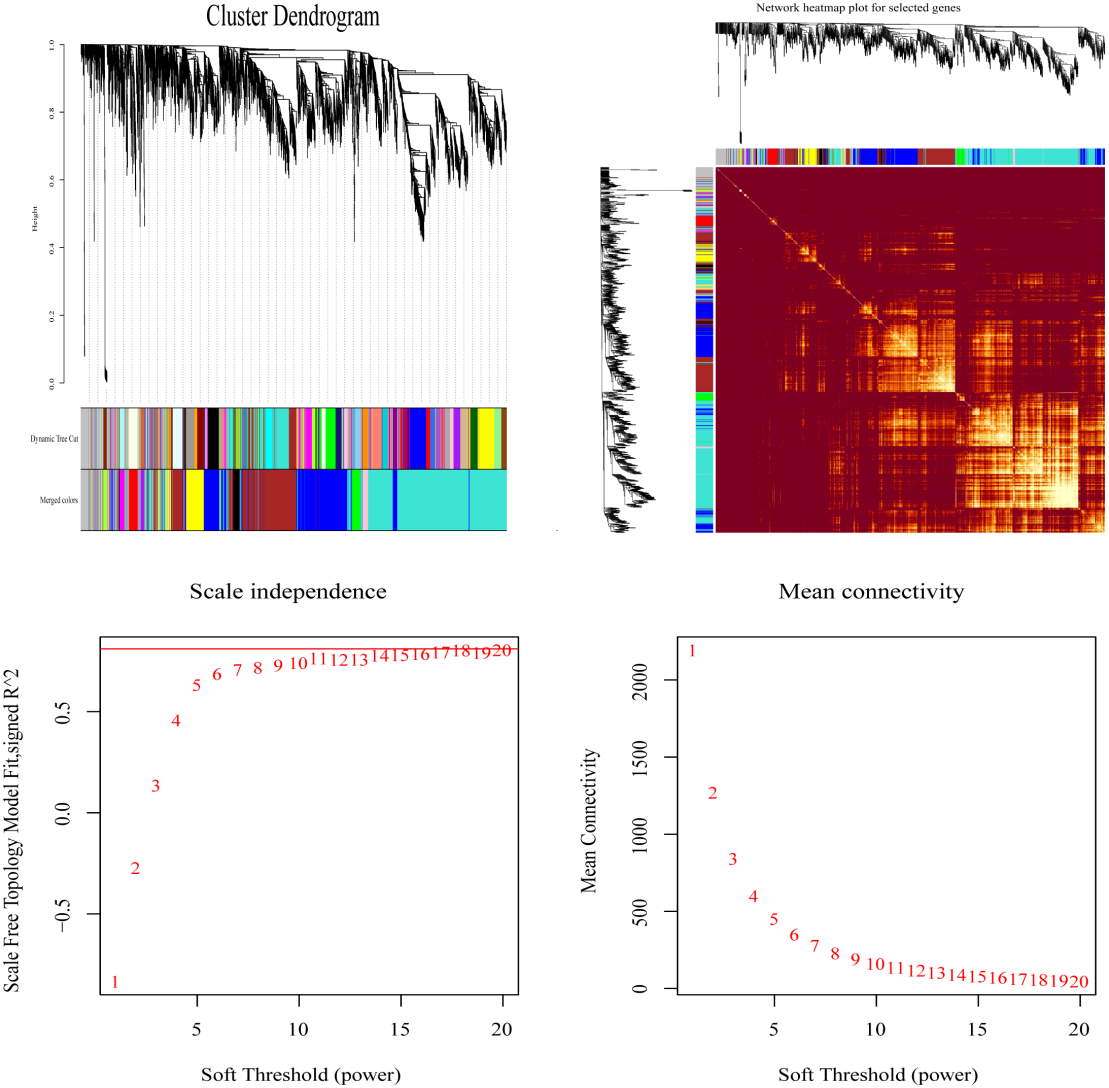
Weighted gene co-expression network analysis (WGCNA) of DEGs (n=5).

**Figure 12:**
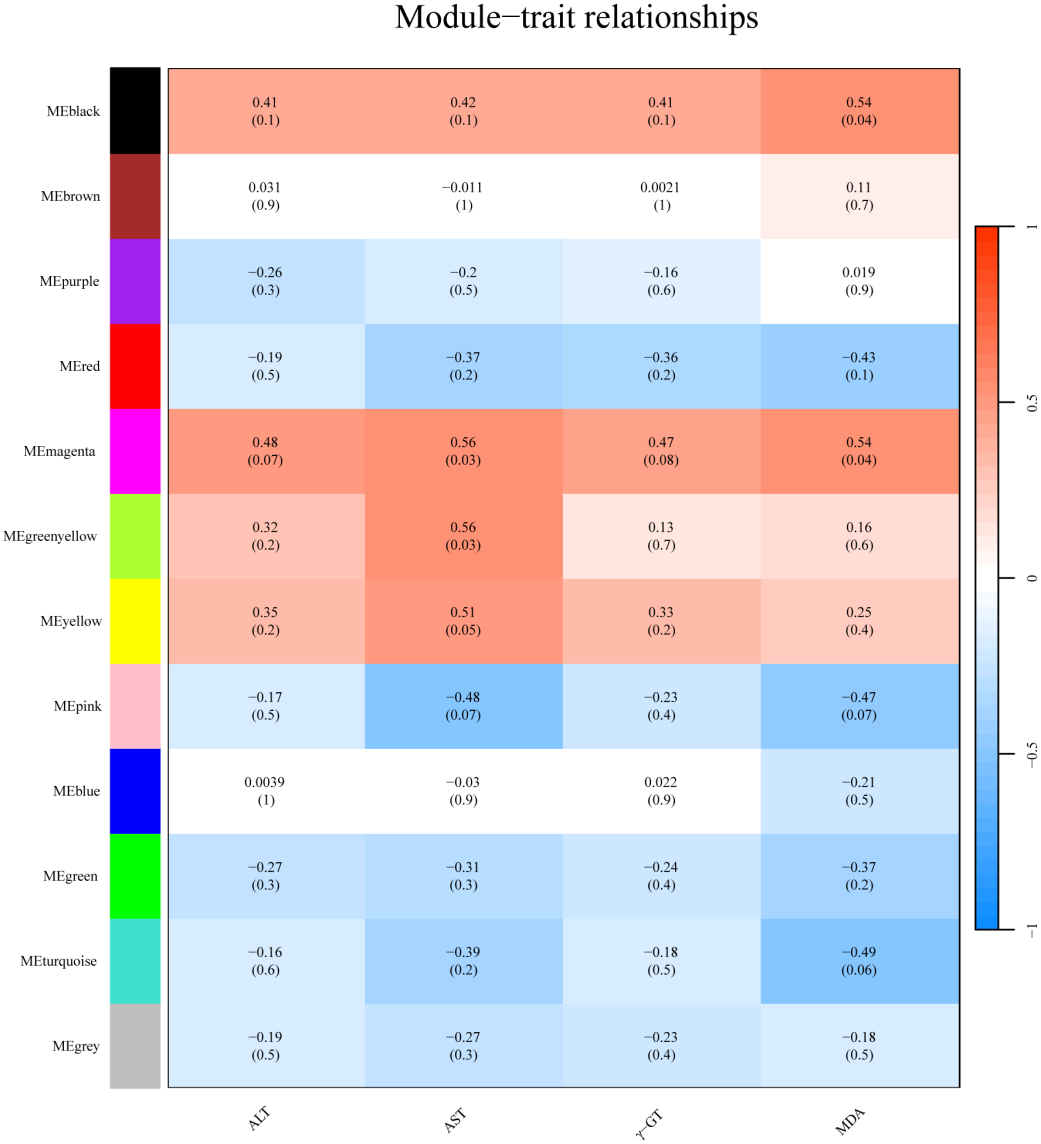
Exploration of hub genes regulating the liver inflammatory response in turbot by genistein using weighted correlation network analysis (n=5).

**Figure 13:**
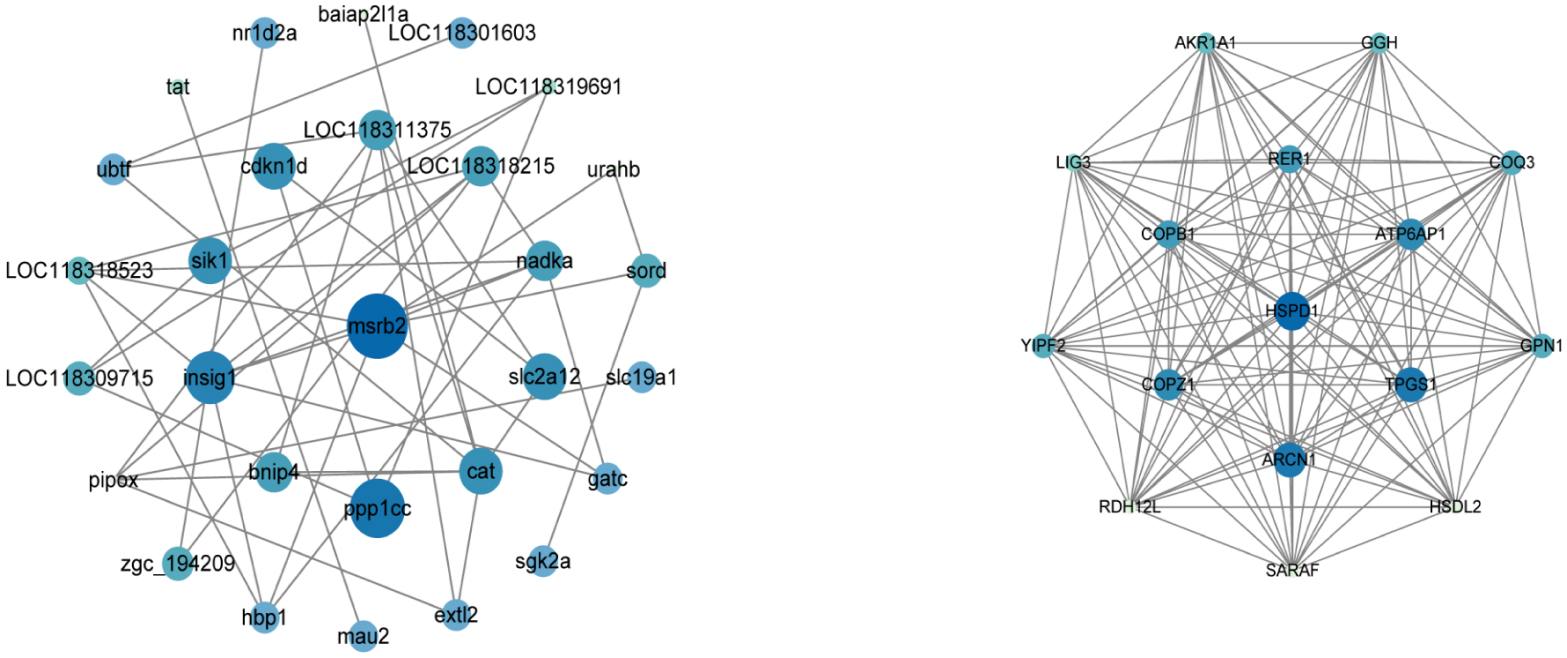
Screening of central genes in turbot liver inflammatory response regulated by genistein using Cytoscape 3.0.

Based on the results of liver transcriptomic analysis, the mechanism by which genistein in oxidized fish oil feed alleviates inflammatory responses in turbot livers remains unclear. Therefore, it was necessary to identify hub genes using WGCNA (**Figures 11**, **12**). The correlation between key liver function-related indicators and the gene modules of each group was analyzed. Results showed that the gene module most strongly correlated with liver function-related indicators was the MEmagenta module. Then, KEGG functional predictions were performed on the MEmagenta module genes. The results showed that the KEGG-enriched signaling pathways associated with inflammation and oxidative damage primarily included Salmonella infection, oxidative phosphorylation, the proteasome, RNA-related pathways, molecular synthesis pathways, cellular transport and protein export pathways, and lipid metabolism pathways. Analysis of the relationships between the MEmagenta module and MEblack gene network nodes suggested that the *msrb2* and *hspd1* (**Figure 13**) were identified as potential hub genes involved in the process by which genistein alleviated the inflammatory and oxidative injury in turbot livers resulting from oxidized fish oil.

## 4 Discussion

The research aimed to assess the defense ability of genistein on oxidized fish oil-elicited liver damage in turbot and to explore the associated molecular mechanisms. In previous research, oxidized fish oil induced hepatic mitochondrial damage in turbot, and dietary genistein supplementation effectively ameliorated such adverse alterations. These findings were consistent with previous reports in teleosts. Studies in catfish (*Pelteobagrus fulvidraco*) have showed that oxidized fish oil triggers oxidative stress, elevates MDA levels, and impairs mitochondrial ultrastructure (Zhang et al., 2021). Likewise, dietary inclusion of lipid peroxide-rich fish oil or thermally oxidized marine oil has been documented to cause mitochondrial damage in largemouth bass (*Micropterus salmoides*) (Yin et al., 2018). Accumulating evidence from mammalian studies has revealed that mice with Sjögren’s syndrome display typical mitochondrial ultrastructural lesions, including mitochondrial membrane rupture, mitochondrial swelling, and loss of mitochondrial cristae. Notably, genistein markedly restore the integrity of mitochondrial cristae by binding to estrogen receptors, upregulating the lncRNA Xist, and repressing the pro-ferroptotic function of acyl-CoA synthetase long-chain family member 4 (ACSL4) (Mao et al., 2024). Furthermore, a study demonstrated that genistein could effectively repair impaired mitochondrial ultrastructure, alleviate mitochondrial swelling and cristae damage, and preserve the integrity of mitochondrial membranes in pathological cellular models. Meanwhile, genistein reduced intracellular ROS accumulation, relieved excessive oxidative stress, stabilized mitochondrial membrane potential, and maintained normal mitochondrial energy metabolism (Diaz et al., 2022).

Moreover, oxidized fish oil feed poses multiple risks to aquaculture fish, such as significantly reducing weight gain rates and feed conversion efficiency, thereby inhibiting growth; furthermore, it could induce lipid peroxidation and damage vital organs such as the liver and intestines (Sana Yagoub Abdallah Tahir and Kop, 2026; Ma et al., 2024; Peng et al., 2017). As a result, the principal purpose of this study was to elucidate the role of dietary oxidized fish oil in modulating oxidative damage and inflammatory reactions in turbot. Results revealed that the mRNA levels of pro-inflammatory cytokines, oxidative stress-related genes, and apoptosis-related genes were strongly elevated in the OFO group, whereas such upregulation was effectively reversed in the GEN40 group, which aligned with earlier findings. Under arsenic stress, the upregulated expression of the apoptotic factor *caspase-3* in Nile tilapia was remarkably reversed by dietary genistein supplementation (Alotaibi et al., 2025). Research regarding common carp (*Cyprinus carpio L.*) has shown that adding genistein to fish oil-enriched feed significantly downregulated the mRNA levels of pro-inflammatory cytokines in the liver (Yang et al., 2021). Analogous findings have been demonstrated in PC12 cells, in which genistein effectively elevated mitochondrial membrane potential (MMP) and decreased intracellular ROS accumulation, therefore attenuating oxidative stress-triggered cell injury (Zhang et al., 2025). Meanwhile, genistein likely alleviated oxidant stress in primary rat cortical neurons by downregulating the mRNA level of NF-κB/p65, inhibiting the phosphorylation of p65 and inhibitor of NF-κB (IκB), and suppressing the activation of the mitogen-activated protein kinase (MAPK) signaling pathway (Qian et al., 2015). Genistein also upregulated Nrf2 and phase II detoxification genes via the extracellular regulated kinase (ERK) and protein kinase C (PKC) signaling pathways, thereby protecting Caco-2 cells against oxidative stress (Zhai et al., 2013). Furthermore, transcriptome profiling uncovered significant enrichment of pathways linked to redox imbalance and immune activation, including PPAR, NOD-like receptor (NLR), peroxisome, and Toll-like receptor (TLR) signaling. Additionally, it has been increasingly demonstrated that genistein mitigates peroxidative condition and hepatic damage through restoration of the Nrf2-HO-1 signaling axis in the liver (Peng et al., 2023). Meanwhile, previous investigations have shown that genistein regulated Nrf2-mediated antioxidant defense and suppress NF-κB-triggered inflammatory responses, thereby ameliorating hepatic injury triggered by the benzotriazole UV stabilizer UV-234 in yellow croaker (Li et al., 2023). Moreover, by activating the GPR30-Nrf2 pathway, genistein quenched ROS and consequently limited NLRP3 inflammasome activation (Cao et al., 2025). However, the absence of significant differences in 4-HNE and 8-iso-PGF2α levels between the OFO,FFO and GEN40 groups may be explained by two possibilities. First, sampling timing is critical, as these peroxidation products are reactive and short-lived; our single end-point measurement may have missed earlier transient elevations or sampled after the peak had already subsided (Folbergrová et al., 2023). Second, the lack of accumulation may reflect a compensatory upregulation of endogenous antioxidant defenses, whereby enzymatic and non-enzymatic systems cooperatively neutralized lipid peroxidation (Sharma et al., 2022).

In addition, this study proposed that *msrb2* and *hspd1* possibly act as the hub genes mediating genistein’s alleviation of oxidized fish oil-induced hepatic oxidative injury and inflammatory reactions in turbot. The MSRB family consists of three members: *msrb1*, *msrb2*, and *msrb3*. The *msrb3* further comprises two subtypes, *msrb3-a* and *msrb3-b*. By reducing and repairing oxidatively damaged methionine residues, these proteins protect various cellular compartments from oxidative damage and maintain overall cellular health. Studies in mammals demonstrated that chondrocytes were provide with protection from H_2_O_2_-induced oxidative stress and apoptosis by MSRB2, whose expression was downregulated in osteoarthritis (OA), potentially through the preservation of mitochondrial function mediated by charged multivesicular body protein 5 (CHMP5) (Huang et al., 2024). The heat shock protein family includes *hsp27*, *hsp40*, *hsp70*, *hsp60*, and *hsp90*, among others (Xie et al., 2026). Meanwhile, *hspd1* is vital for the support of mitochondrial health and metabolic homeostasis, while also participating in an array of key biological processes, including cellular stress, apoptosis regulation, and immune responses. For example, oxidative damage in mouse cells can increase the expression of the *hspd1* gene. Furthermore, the *hspd1* protein responds to oxidative stress and oxidative damage in the body by interacting with proteins such as sirtuin 1 (*sirt1*) within the cell (Li et al., 2024). Earlier findings have corroborated the critical roles of *msrb2* and *hspd1* in attenuating inflammatory responses in mammals; however, research in aquatic animals remains limited, and the molecular mechanisms underlying the functions of *msrb2* and *hspd1* require further investigation. What’ more, additional experiments for functional characterization and mechanistic investigation were scheduled to be performed in our future studies.

## 5 Conclusion

In summary, adding genistein to oxidized fish oil feed effectively reduced redox imbalance and immune-inflammatory activation in the livers of turbot, thereby reducing economic losses during aquaculture. Furthermore, *msrb2* and *hspd1* possibly act as key genes involved in genistein’s mitigation of inflammatory responses in fish livers. Nevertheless, the underlying molecular basis warrants further investigation.

## Data Availability

The datasets presented in this study can be found in online repositories. The names of the repository/repositories and accession number(s) can be found below: https://www.ncbi.nlm.nih.gov/, PRJNA1457694.
