# Supplementary material for: Transcriptomic analysis revealed the protective role of genistein against oxidized fish oil-induced oxidative stress and inflammatory responses in turbot (Scophthalmus maximus)
Source: Front Physiol. 2026 Aug 4;17:1900021. doi: 10.3389/fphys.2026.1900021 (PMC13481184; doi:10.3389/fphys.2026.1900021)
Supplement: Supplementary file 1 [file DataSheet1.pdf]

Supplementary Material

Table 1

Formulation and proximate analysis of experimental diets (g/kg dry matter).

Raw material	Experimental diets		
	FFO	OFO	GEN40
Fishmeal[a]	500.00	500.00	500.00
Peanut meal[b]	100.00	100.00	100.00
Wheat gluten flour[c]	80.00	80.00	80.00
Corn gluten meal[d]	50.00	50.00	50.00
Wheat meal[e]	100.00	100.00	100.00
a-Starch[f]	56.00	56.00	55.96
Fresh fish oil	48.00	0.00	0.00
Oxidized fish oil	0.00	48.00	48.00
soya bean lecithin	20.00	20.00	20.00
Genistein	0.00	0.00	0.04
VC phosphate ester	0.50	0.50	0.50
Vitamin and mineral premix[g]	15.00	15.00	15.00
Sodium alginate	1.00	1.00	1.00
Choline chloride	3.00	3.00	3.00
Antiseptic[h]	1.00	1.00	1.00
Ethoxyquin	0.50	0.50	0.50
Attractants[i]	5.00	5.00	5.00
Squid offal powder[j]	10.00	10.00	10.00
Taurine	5.00	5.00	5.00
Ca(H2PO4)2Calcium phosphate monobasic	5.00	5.00	5.00
Proximate analysis (%/drymatter)			
Crude protein	516.80	504.50	513.90
Crude lipid	113.40	106.00	110.70
Moisture	43.00	46.10	38.90

[a] Fish meal (88.06% dry matter (DM), 70.20% crude protein (CP), 7.50% crude fat (CL)).

[b] Peanut meal (80.39% DM, 54.50% CP, 0.50% CL).

[c] Wheat gluten flour (90.69% DM, 70.50% CP, 0.30% CL).

[d] Corn protein meal (91.91% DM, 62.10% CP, 1.60% CL).

[e] Wheat meal (85.38% DM, 13.70% CP, 0.40% CL).

[f] α-starch (96.61% DM, 0.20% CP, 0.10% CL).

[g] Vitamin and mineral premix (per kg of premix): Vitamin A acetate, 675,000 IU; Vitamin D, 3,150,000 IU; DL-α-tocopheryl acetate, 6 g; Pyridoxine hydrochloride, 1.2 g;Thiamine nitrate, 0.9 g; Riboflavin, 1.35 g; Pyridoxine hydrochloride, 1.05 g; Cyanocobalamin, 0.0075 g; Calcium D-pantothenate, 4.5 g; Nicotinamide, 6.75 g; Folic acid, 0.375 g;d-biotin, 0.015 g; l-ascorbic acid-2-phosphate (calculated as l-ascorbic acid), 15 g; inositol, 10 g; iron (ferrous sulfate), 20 g; zinc (zinc sulfate), 9.6 g; manganese (manganese sulfate), 5 g; Copper (copper sulfate), 0.6 g; Cobalt (cobalt chloride), 0.08 g; Selenium (sodium selenite), 0.04 g; Iodine (calcium iodate), 0.08 g; Zeolite powder, etc., moisture content ≤10%.

[h] Antiseptic, calcium propionate: bile acid = 1:1.

[i] Attractant,: DMPT: glycine: alanine: inosine-5-diphosphate trisodium salt = 4:2:2:1.

[j] Squid offal powder (80.73% DM, 7.20% CP, 0.90% crude lipids) were purchased from Shandong Haiding Agriculture and Animal Husbandry Co., Ltd. (Jinan, China).

Table 2

Statistic summary of RNA-Seq data (n=5).

Group	Raw reads (item)	Raw bases (G)	Clean reads (item)	Clean bases (G)	Error rate (%)	Q20 (%)	Q30 (%)	GC pct (%)
FFO1	47240206	7.09G	46452094	6.97G	0.01	99.16	96.12	49.05
FFO2	47870366	7.18G	47125598	7.07G	0.01	99.14	96.16	46.99
FFO3	49833088	7.47G	49145762	7.37G	0.01	99.2	96.32	47.91
FFO4	43742712	6.56G	42967534	6.45G	0.01	99.27	96.55	47.92
FFO5	45348168	6.8G	44705238	6.71G	0.01	99.15	96.11	50.01
OFO1	49058180	7.36G	48291686	7.24G	0.01	99.11	95.92	49.6
OFO2	43457146	6.52G	42769802	6.42G	0.01	99.11	95.97	49.83
OFO3	43286784	6.49G	42409406	6.36G	0.01	99.15	96.06	51.48
OFO4	41471262	6.22G	40726160	6.11G	0.01	99.14	96.12	49.46
OFO5	43151136	6.47G	42517000	6.38G	0.01	99.32	97.19	47.83
GEN1	46878110	7.03G	45958426	6.89G	0.01	99.18	96.29	48.35
GEN2	47073634	7.06G	46189840	6.93G	0.01	99.23	96.37	48.79
GEN3	47272044	7.09G	46463658	6.97G	0.01	99.19	96.21	50.29
GEN4	60378894	9.06G	58701222	8.81G	0.01	99.11	96.05	47.99
GEN5	44557578	6.68G	43813954	6.57G	0.01	99.08	96.02	46.98

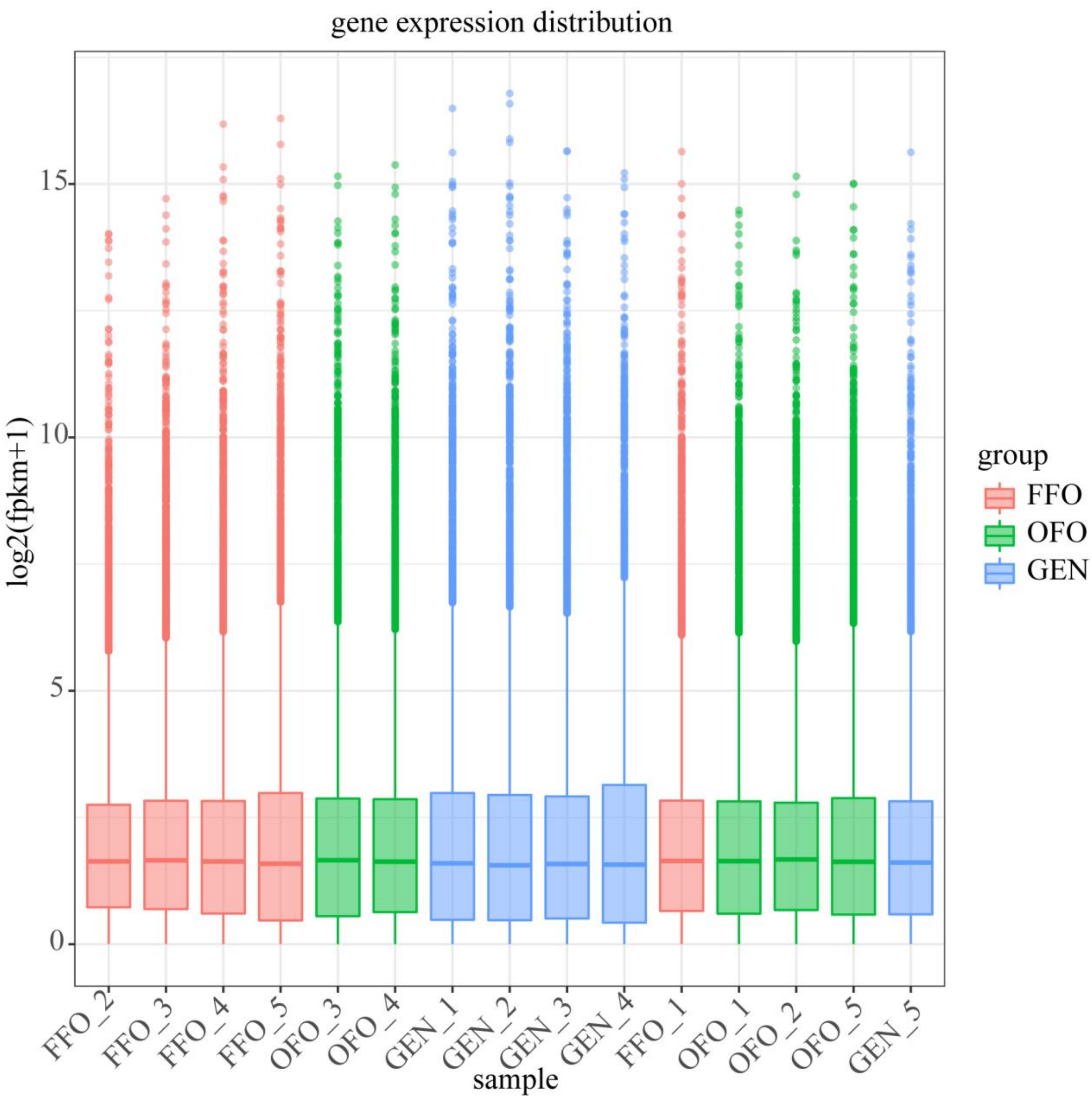

Fig. 1. Distribution of transcriptome gene expression (n=5).

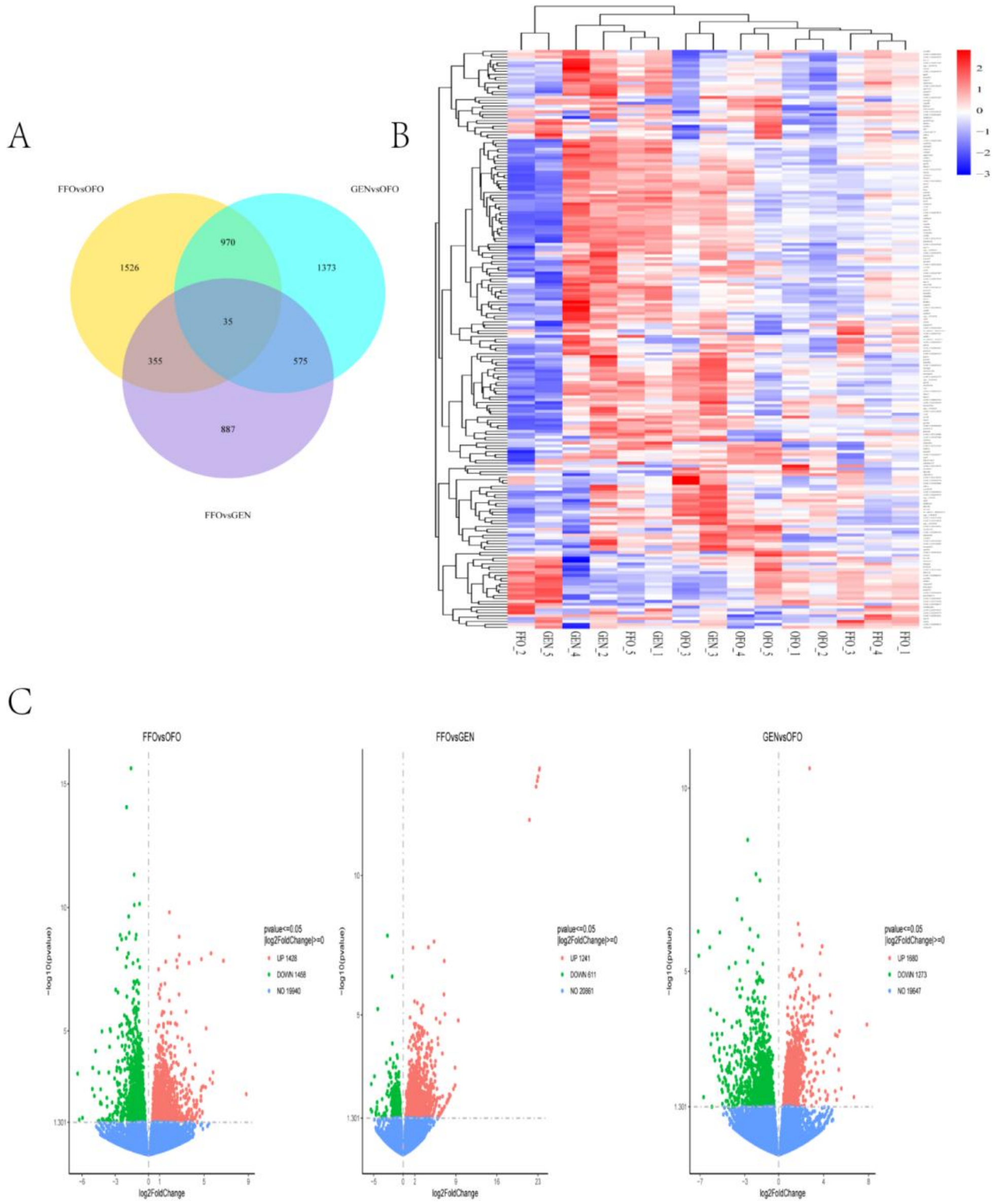

Fig. 2. Identification of differentially expressed genes (DEGs) among the FFO, OFO, and GEN40 groups (n = 5 per group). (A) Venn diagram showing the number of unique and shared DEGs among the three dietary treatment groups. (B) Cluster heatmap displaying the expression profiles of DEGs across all individual samples; red and blue indicate high and low relative expression levels, respectively (Z-score normalized). (C) Volcano plots for each pairwise comparison (FFO vs OFO, FFO vs GEN40, and OFO vs GEN40). Red and blue dots represent significantly up-regulated and down-regulated genes.
